# Associated Targets of the Antioxidant Cardioprotection of *Ganoderma lucidum* in Diabetic Cardiomyopathy by Using Open Targets Platform: A Systematic Review

**DOI:** 10.1155/2020/7136075

**Published:** 2020-07-25

**Authors:** Fahmi Shaher, Hongbin Qiu, Shuqiu Wang, Yu Hu, Weiqun Wang, Yu Zhang, Yao Wei, Hisham AL-ward, Mahfoudh A. M. Abdulghani, Sattam Khulaif Alenezi, Salem Baldi, Shaobo Zhou

**Affiliations:** ^1^Department of Pathophysiology, College of Basic Medicine, Jiamusi University, Jiamusi, China; ^2^Department of Physiology, College of Basic Medicine, Jiamusi University, Jiamusi, China; ^3^Department of Pharmacology, College of Pharmacy, Jiamusi University, Jiamusi, China; ^4^Department of Biochemistry and Molecular Biology, College of Basic Medicine, Jiamusi University, Jiamusi, China; ^5^Department of Pharmacology and Toxicology, Unaizah College Pharmacy, Qassim University, Saudi Arabia; ^6^Department of Clinical Laboratory Diagnostics, College of Basic Medicine, Dalian Medical University, China; ^7^School of Life Sciences, Institute of Biomedical and Environmental Science and Technology, University of Bedfordshire, Luton LU1 3JU, UK

## Abstract

Even with substantial advances in cardiovascular therapy, the morbidity and mortality rates of diabetic cardiomyopathy (DCM) continually increase. Hence, a feasible therapeutic approach is urgently needed. *Objectives*. This work is aimed at systemically reviewing literature and addressing cell targets in DCM through the possible cardioprotection of *G*. *lucidum* through its antioxidant effects by using the Open Targets Platform (OTP) website. *Methods*. The OTP website version of 19.11 was accessed in December 2019 to identify the studies in DCM involving *G*. *lucidum*. *Results*. Among the 157 cell targets associated with DCM, the mammalian target of rapamycin (mTOR) was shared by all evidence, drug, and text mining data with 0.08 score association. mTOR also had the highest score association 0.1 with autophagy in DCM. Among the 1731 studies of indexed PubMed articles on *G. lucidum* published between 1985 and 2019, 33 addressed the antioxidant effects of *G. lucidum* and its molecular signal pathways involving oxidative stress and therefore were included in the current work. *Conclusion*. mTOR is one of the targets by DCM and can be inhibited by the antioxidative properties of *G. lucidum* directly via scavenging radicals and indirectly via modulating mTOR signal pathways such as Wnt signaling pathway, Erk1/2 signaling, and NF-*κ*B pathways.

## 1. Introduction

Cardiovascular complications are associated with diabetes and lead to high mortality [1, 2]. Diabetic cardiomyopathy (DCM) is one of the main causes of heart injury and death in patients with diabetes. A total of 1.6 million deaths worldwide are directly attributed to diabetes every year [3]. Independent of coronary artery disease, DCM has increased prevalence during the last two decades and is experienced by 55% of patients with diabetes [4]. With diabetes being a global epidemic, the number of patients with DCM has increased. For the last two decades, the number of people with diabetes worldwide has increased from 151 million in 2000 to 425 million in 2017 and is estimated to increase to 629 million by 2045 [5]. The risk of developing DCM is higher for patients with diabetes than that for those without diabetes [6] and increases 2 to 4 times for those with more than a 10-year span of diabetes [7, 8]. Once DCM has developed, reducing its morbidity and mortality is difficult even with pharmacological improvement in terms of regulating blood glucose and insulin sensitivity. Clinical and preclinical investigations have examined the complexity of the pathophysiological consequences of DCM.

Clinical studies in patients with DCM reported that the pathological remodeling of the heart, which is characterized by left ventricular concentric hypertrophy and perivascular and interstitial fibrosis commencing to diastolic dysfunction and extended contraction and relaxation [9, 10], shortens ventricular ejection and increases wall stiffness [11, 12]. The influence of the diabetic condition on heart and cardiomyocyte function has been experimentally evaluated.

DCM and cardiac dysfunction are initiated in diabetic-induced experimental animals from 2 to 12 weeks [13]. Streptozotocin-induced diabetes in mice leads to the morphological changes of heart tissues, interstitial collagen deposition, cardiac hypertrophy, fibrosis, and remarkable elevation of paracrine of angiotensin II level in myocardium and NADPH oxidase activities, which are considered the primary source of free radicals in the cardiomyocytes of diabetic heart [14]. Connective tissue growth factor mediates cardiac fibrosis in diabetes [13, 15]. In diabetic mice with cardiomyopathy, the expression of sarcoplasmic reticulum calcium ATPase and [Ca^+2^] ion transient is reduced [16]. Sarcoplasmic reticulum calcium ATPase is a primary cardiac isoform of calcium pump transporting calcium from cytoplasm to sarcoplasmic reticulum during diastolic relaxation [17].

Even with substantial advances in cardiovascular therapy, diabetic morbidity and mortality rate are continually increasing, and a feasible therapeutic approach for DCM is still lacking. Exploring the medication targets for DCM may further identify novel drugs and improve specific therapies for DCM. Therapeutic targets for DCM with natural resources are considered as one of the main reservoirs for drug discovery. Therefore, novel therapeutics for a range of targets must be developed to prevent DCM progression. This study identifies molecular target involvement and its association with DCM by using the Open Targets Platform (OTP) website established by Biogen, EMBL European Bioinformatics Institute, GlaxoSmithKline, and Wellcome Trust Sanger Institute. The OTP provides comprehensive and up-to-date data for drug molecular targets associated with relative diseases. Oxidative stress (OS) may be a key factor in the molecular and cellular mechanisms of diabetes-induced DCM [18]. Hence, targeting OS-related processes could be a promising therapeutic strategy for DCM.

*Ganoderma (G.) lucidum*, which is known in Chinese as “Lingzhi,” is a medicinal mushroom commonly used as a Chinese herbal medicine and the main ingredient in many conventional combinations or dietary supplements [19]. This name has been proposed by Petter Adolf Karsten from England in the late 19^th^ century and has been applied in various places such as Asia, Africa, Oceania, and Europe [20]. Lingzhi has been widely cultivated in China and has a long history as a traditional Chinese medicine. Chinese *G. lucidum* exhibits high variability of basidioma morphology and more or less consistency in its microscopic characters, e.g., short clavate cutis elements, Bovista-type ligative hyphae, and strongly echinulated basidiospores [21]. *G. lucidum* also contains various bioactive compounds, such as flavonoids, ganoderic acid, phenolics, and polysaccharides [21], that can treat many chronic diseases including diabetes and its complications by counteracting OS. Preclinical studies reported the beneficial effects of *G. lucidum* against OS-induced diseases, its liver protection against CCl_4_-induced OS [22], skin protection against croton oil-induced lipid peroxidation in mice [23], and thymus and spleen protection against 5-fluorouracil-induced OS in mice [21]. This systematic review is aimed at discussing the potential cell targets and cardioprotective pathway of *G. lucidum* based on preclinical and clinical investigations.

## 2. Methods

The OTP website version 19.11 (OTP V 19.11) was used to prioritize and identify the targets associated with DCM. The OTP provides score and rank target-disease associations and integrates evidence from six resources, including genetics, genomics, transcriptomics, drugs, animal models, and scientific literature [24, 25]. Two main steps of searching were performed in December 2019. In the first step, the term “diabetic cardiomyopathy” was used, and all the targets associated with DCM were listed according to available evidence recorded through bioinformatic processing, including data evidence of drug, text mining, genetic association, somatic mutation, pathways and signals, RNA signal, and animal model. The resulting targets with the highest association with DCM from the first step were used to further search for evidence on *G. lucidum* cardioprotection.

**Figure 1:**
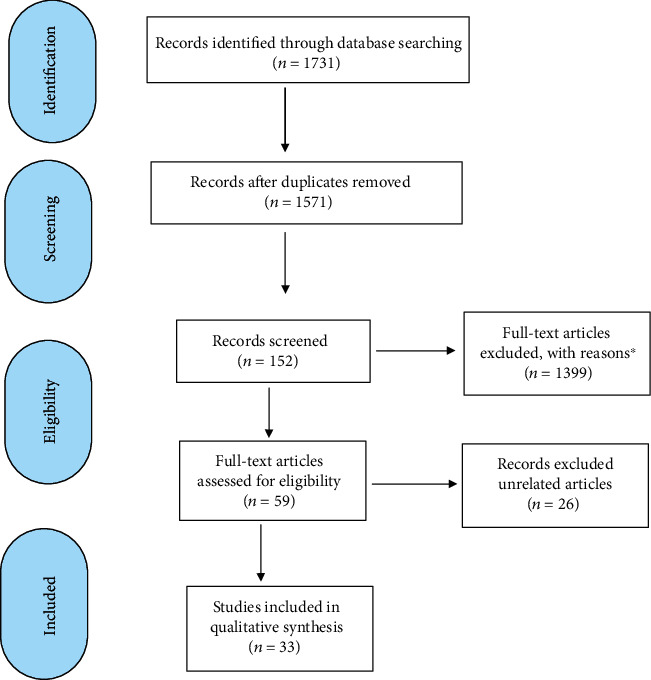
A PRISMA flow diagram summarising the study selection process. Antioxidant of *G. Lucidum*; PRISMA: Preferred Reporting Items for Systematic Reviews and Meta-Analyses. ^∗^After exclusion of other antioxidant activity studies of *G. Lucidum.*

This systematic review on the antioxidant activity of *G. lucidum* was described as follows. Abstracts published from 1985 to July 2019 were reported as guided by the Preferred Reporting Items for Systematic Reviews and Meta-Analyses [26] (Figure 1). The key terms used were *G. lucidum* and spore of *G. lucidum*. In this step, the studies were divided into seven 5-year periods to easily read and select related abstracts. The search was limited to studies published in English and Chinese languages. Inclusion criteria were as follows: studies must focus on (1) *G. lucidum* and its (2) antioxidant, antidiabetic, and cardioprotective activities. Exclusion criteria were as follows: studies focusing on (1) mushrooms other than *G. lucidum* and (2) not related to its antioxidant activities such as the botanical and genetic studies of *G. lucidum.*

## 3. Results

### 3.1. Targets Associated with DCM in Diabetes Integrated by OTP

#### 3.1.1. DCM and Its Associated Targets

A total of 309 targets were associated with DCM based on evidence from drug and text mining data with overall association scores from 0.004 to 0.177 (Table 1, supplementary file (available here)). Among the selected drug data, only two targets, namely, carnitine palmitoyltransferase 1B (CPT1B) and 2 (CPT2) were associated with DCM with 0.1 score association. A total of 306 targets were identified from text mining. Only the mechanistic target of rapamycin kinase (mTOR) was common in both types of data. A total of 309 targets were expressed in 32 tissue organs including the heart and were involved in 19 pathway types (Table 2, supplementary file (available here)). Among these 309 targets, 155 were expressed in the heart tissues with overall association scores ranging from 0.007 to 0.177 (Table 1). Among the 19 pathways, 4 targets were included in autophagy (Table 2), namely, mTOR, beclin 1 (BECN1), parkin RBR E3 ubiquitin protein ligase (PRKN), and voltage-dependent anion channel 1 (VDAC1) with scores of 0.1, 0.06, 0.05, and 0.03, respectively.

mTOR was further investigated, and its association with heart diseases ranged from 0.0004 to 0.8588, which is the overall association score for 49 subtypes of heart diseases. mTOR had 0.1 and 0.8 overall association scores with DCM and hypertrophic cardiomyopathy, respectively (Table 3).

**Figure 2:**
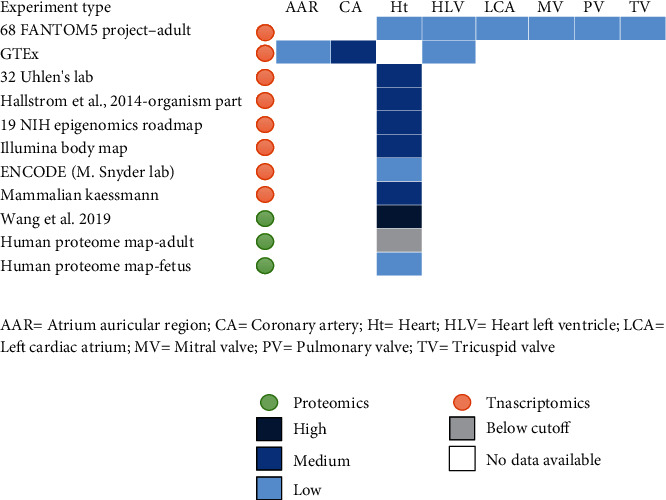
mRNA and protein baseline expression of mTOR in the heart.

mTOR is a serine/threonine-protein kinase playing as a central regulator of cellular metabolism, growth, and survival in response to hormone growth factor [27], nutrients, energy, and stress signals [28, 29]. According to UniPort, mTOR can be found in different subcellular locations including the membranes of endoplasmic reticulum, Golgi apparatus, outer mitochondrion, microsome, and lysosome; lysosome, cytoplasm, nucleus, and PML nuclear body. The RNA and protein expression levels of mTOR are present in several organs including the heart, e.g., the medium RNA and high protein levels of mTOR are expressed in the left ventricle, atrium, and coronary artery but not in the heart muscles (Figure 2).

#### 3.1.2. Evidence on the Cardioprotection of *G. lucidum*

**Figure 3:**
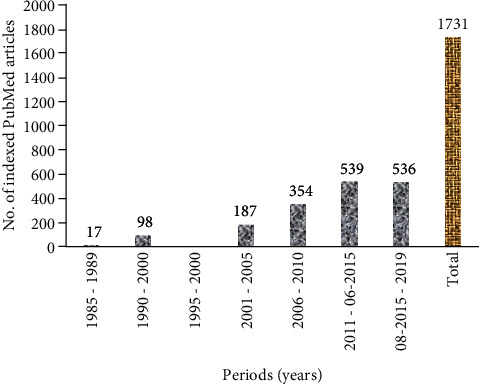
Number of studies on *G*. *lucidum* during 1985-2019.

A total of 1731 articles were identified (Figure 3) and further divided into seven 5-year time periods. The first period ranged from 1985 to 1989, and the last period ranged from August 2018 to August 2019 (Figure 1). These articles were reviewed in the following three phases. First, 1571 articles remained after the duplicated ones were removed. Second, articles that did not satisfy the inclusion criteria based solely on their titles (remaining 1399 articles) and abstracts (remaining 59 articles) were excluded. Lastly, the remaining articles were scanned, and those that did not meet our inclusion criteria were excluded. After the initial screening of titles and abstracts, the 59 remaining articles were screened for the second time by two individual reviewers. Inclusion of full articles was agreed upon by two reviewers prior to data extraction. Finally, 33 studies were considered eligible for the review (Figure 2). In this section, the collected pieces of evidence were divided into two main parts, namely, the in vivo antioxidant of *G. lucidum* (14 studies, Table 4), in which the in vivo effect of antioxidant on the parameters related to OS was discussed, and the in vitro antioxidant of *G. lucidum* (19 studies, Table 5), in which the in vitro effect of antioxidant activities and possible molecular mechanisms was elaborated.

### 3.2. *In Vivo* Antioxidant Activity and Protective Effect of *G. lucidum*

According to 10 in vivo experimental studies, *G. lucidum* has antioxidant activities and protects against OS through four main factors in different tissues, such as the heart, liver, thymus, spleen, eyes, and skeletal muscles, and by regulating chemical-level OS parameters in blood circulation (Table 4). *G. lucidum* exhibits its antioxidant effects by increasing the antioxidant enzymes and inhibiting the enzymes involved in OS. *G. lucidum* also increases the activities of superoxide dismutase (SOD), glutathione-S-transferase (GST), glutathione peroxidase (GPx), catalase (CAT), mitochondrial succinate dehydrogenase (SDH), and Mn-SOD and reduces glutathione (GSH) levels. By contrast, *G. lucidum* decreases the activities of nitric oxide synthase (NOS), cytochrome P450 2E1 (CYP2E1), xanthine oxidase (XOD), and myeloperoxidase (MPO). *G. lucidum* also significantly decreases lipid peroxidation levels, advanced oxidation protein products (AOPPs), and malondialdehyde (MDA) levels.

The first factor is the four toxic substances, including CCl_4_-induced oxidative stress (OS) in the liver, croton oil produced OS in the skin through inflammation, N-methyl-N-nitrosourea (MNU) causing retinal photoreceptor cell lesions in the eyes, and 5-fluorouracil-induced OS in the thymus and spleen of mice. Oral administration of *G. lucidum* polysaccharides (GLPs) represses free radical lipid peroxidation induced by CCl_4_ to reduce the enzyme activities of NOS and CYP2E1. Significant inhibition of NOS and CYP2E1 activities and MDA and IL-1*β* levels was noted in liver tissues, and depleted levels of interleukin- (IL-) 1*β*, IL-18, IL-6, and tumor necrosis factor-*α* were found in serum. In CCl_4_-induced liver damage, highly reactive trichloromethyl free radicals are generated by the cytochrome P450 isozymes (P450s) of the endoplasmic reticulum [22]. Topical administration of *G. lucidum* ethanol extract inhibits the croton oil-induced lipid peroxidation in the skin of mice [23]. Ganoderma spore lipid (GSL) shows a protective effect on MNU-induced retina injury by inhibiting the related apoptosis to modulate the expression levels of Bax, Bcl-xl, and caspase-3 [30]. GLPs also exhibit an antioxidant effect in 5-fluorouracil-induced OS and improve SOD, an intracellular compound that protects against oxidative processes initiated by superoxide anion and GPx contents in the spleen and thymus of mice [31].

The second factor creates conditions in biological systems that can induce OS, such as exercise-like exhaustive swimming, which is OS induced in skeletal muscles, and a carotid artery ligation, which disturbs the flow-induced OS level of manganese-dependent superoxide dismutase (Mn-SOD) in blood vessels. GLPs show protective effects against comprehensive swimming-induced OS by improving the activities of antioxidant enzymes (SOD, GPx, and CAT) and decreasing the MDA levels in the skeletal muscle of mice [32]. Oral ganoderma triterpenoids (GTs) protect against disturbed flow-induced OS through carotid artery ligation, which leads to chronic OS and inflammation that are features of early atherogenesis in mice, and by preventing neointimal thickening 2 weeks after ligation. Early atherogenesis includes neointimal hyperplasia and endothelial dysfunction due to flow turbulence in the ligated artery as induced by OS. GTs alleviate OS and restore the atheroresistent status of endothelium by inhibiting endothelin-1 induction, von Willebrand factor, and monocyte chemoattractant protein-1 after 3-day ligation as atherogenic factors [33]. Inflammatory cytokines, OS-induced endothelial dysfunction, and chronic OS contribute to endothelial impairment and induces atherogenesis.

The third factor in OS includes diseases such as type II diabetes mellitus (DM) and cancer. In type II DM, the beneficial effects of *G. lucidum* on abnormal heart and testis and epididymal cells of rats with streptozotocin-induced type II DM were evaluated. GLPs improve the myocardial ultrastructure by reducing MDA, activating antioxidant enzymes (GSH-Px, CAT, SOD, and NO) in cardiac tissues, and reducing lipid peroxidation in type II DM rats [34]. *G. lucidum* spores protect the testis of rats with type II DM by substantially increasing the mitochondrial SDH and decreasing the activities of XOD and MPO [35]. *G. lucidum* spores protect epididymal cells and counteract their apoptosis that damages the mitochondria and disequilibrium of calcium homeostasis by reducing the amount of mitochondrial cytoplasm cytochrome C in type II DM rats [36]. GLP administration enhances the immunity and antioxidant activities in N-methyl-N9-nitro-nitrosoguanidine-induced gastric cancer in Wistar rats. GLP remarkably reduces the levels of serum IL-6 and TNF-*α* and increases the levels of serum IL-2, IL-4, and IL-10. In addition, GLP improves the levels of SOD, CAT, and GSH-Px in serum and gastric tissues [37].

The fourth factor involved in OS is aging. *G. lucidum* administration ameliorates the age-related decline of antioxidant status in aged mice, substantially elevates the activities of GST, Mn-SOD, GPx, and CAT, and reduces GSH. By contrast, lipid peroxidation, AOPP, and reactive oxygen species (ROS) are reduced [38] (Table 4).

### 3.3. *In Vitro* Antioxidant of *G. lucidum* and Its Possible Pathway

Chemical antioxidant tests consistently revealed the free radical scavenging activity of *G. lucidum*. Twelve studies reported the scavenging activity of *G. lucidum* for different free radicals including 2,2-diphenylpicrylhydrazyl radical (DPPH^·^), 2,2′-azino-bis(3-ethylbenzthiazoline-6-sulphonic acid) radical (ABTS^·^+), hydroxyl radical (HO^·^), and hydrogen peroxide radicals (H_2_O_2_) [39–49] (Table 5). *G. lucidum* also inhibits lipid peroxidation [23, 49]. In some studies, *G. lucidum* protects against DNA damage [41, 42, 50]. The results of chemical antioxidant tests regarding the antioxidant properties of *G. lucidum* are also in agreement with the cell-based antioxidant assays. *G. lucidum* shows free radical scavenging activity for H_2_O_2_ in RAW264.7 cells incubated with *G. lucidum* lipopolysaccharide and protects against H_2_O_2_-induced cell death [48]. *G. lucidum* also hinders sphingomyelinase activity in incubated RAW264.7 cells with lipopolysaccharide [51]. In addition, *G. lucidum* prevents lipid peroxidation in two cell models, namely, WBCs incubated with lipopolysaccharide to induce OS [39] and hepatocytes incubated with CCl_4_ to induce OS [52]. In both cell models, *G. lucidum* showed protection by elevating the antioxidant enzyme activity (SOD, GPx, and GR) and improving the GSH level. Moreover, *G. lucidum* protects macrophages in human monocytic cells incubated with lipopolysaccharide to stimulate NO production [53].

Wnt, Erk1/2, and NF-*κ*B are the possible signaling pathways of *G*. *lucidum* that support its antioxidant and protective effects. A pancreatic cell study suggested *β*-catenin in the Wnt signaling pathway as a target of ganoderic acid A, thus leading to cell protection and effective scavenging of ROS [54]. The Wnt signaling pathways transfer the signals from extracellular to intercellular and are stimulated by the Wnt protein binding to the cytoplasmic family receptor, which occurs in downstream cell signaling and controls the transcription of genes. In the canonical Wnt pathway, *β*-catenin accumulates in the cytoplasm and is further translocated into the nucleus, and this phenomenon is widely recognized as a regulation marker of fat and glucose metabolism and *β*-catenin/Wnt signaling involved in insulin secretion [54]. In 2006, Thyagarajan and his colleagues mentioned that *G. lucidum* modulates Erk1/2 signaling and transcription factors AP-1 and NF-*κ*B and downregulates c-Fos, whose expression can be induced by OS as the result of the inhibited OS-induced invasive behavior of breast cancer cells. A high H_2_O_2_ concentration (5 mM) can stimulate Erk1/2 signaling in MCF-7 cells [55].

In addition to its antioxidant activities, *G. lucidum* also exhibits an anti-inflammatory property and modulates the immune system. It can reverse LPS-induced inflammation by downregulating inflammatory mediators such as NF-*κ*B, thus substantially inhibiting NOS and reducing NO level [39]. *G. lucidum* also modulates the immune system byregulating cytokine production in RAW264.7 macrophages [56, 57]. Moreover, it increases the formation of autophagosomes and controls proteins (Vps34, beclin 1, LC3-I, LC3-II, and p62) that induce autophagy in a gastric adenocarcinoma cell line. *G. lucidum* increases the cellular levels of LC3-II and decreases the cellular levels of p62 (Table 5).

## 4. Discussion

Among the 155 targets associated with DCM, mTOR, CPT1B, and CPT2 have the highest association. mTOR acts as a core regulator of cellular metabolism, growth, and survival in response to hormone growth factors, nutrients, energy, and stress signals. An animal study confirmed that streptozotocin-induced diabetes increases mTOR levels in rats [58]. mTOR can be found in different cellular locations including membrane, cytoplasm, and nucleus and different cellular organs (mitochondria, Golgi, and endoplasmic reticulum) and therefore is involved directly or indirectly in regulating the phosphorylation of at least 800 proteins (OPT.V19.11). mTOR functions through two distinct signaling complexes of mTORC1 and mTORC2 [59]. When activated, mTORC1 upregulates protein synthesis by phosphorylating the key regulators of mRNA translation and ribosome synthesis. mTORC1 also regulates protein synthesis [29], lipid synthesis [60], and mitochondrial biogenesis and stimulates the pyrimidine biosynthesis pathway through acute and delayed regulations. In acute regulation, mTORC1 stimulates pyrimidine biosynthesis through the ribosomal protein S6 kinase B1-mediated phosphorylation of biosynthetic enzyme carbamoyl-phosphate synthetase 2, aspartate transcarbamylase, and dihydroorotase; these enzymes catalyze the first three steps in de novo pyrimidine synthesis [61]. In delayed regulation, mTORC1 stimulates pyrimidine biosynthesis through the transcriptional enhancement of the pentose phosphate pathway, which produces 5-phosphoribosyl-1-pyrophosphate, an allosteric activator of pyrimidine biosynthesis enzyme at a later step in the synthesis. In addition, mTORC1 regulates ribosome synthesis by activating RNA polymerase III-dependent transcription through the phosphorylation and inhibition of MAF1 protein, a RNA polymerase III-repressor. When nutrients are available and mTOR kinase is active, MAF1 is hyperphosphorylated, and RNA polymerase III is engaged in the transcription [62]. Stress-induced MAF1 dephosphorylation resulted in nuclear localization, increased targeting of gene-bound RNA polymerase III, and decreased transcriptional readout [63, 64]. Moreover, mTORC1 is involved in the negative feedback regulation of autophagy on upstream growth factor signaling during microtubule regulation [64–66].

mTORC2 regulates other cellular processes such as survival and organization of cytoskeleton, actin cytoskeleton [67], osteoclastogenesis, and circadian clock function. In a pressure-overloaded male mouse heart, mTORC2 maintains a contractile function [68]. In brown adipose tissues, mTOR complex 2 has a role in *β*3-adrenoceptor-stimulated glucose uptake by stimulating the translocation of newly synthesized GLUT1 to the plasma membrane, thereby increasing the glucose uptake [69]. mTOR complex 2 regulates the proper turnover of insulin receptor substrate-1 [70].

*G. lucidum* exhibits cardiac protection via its antioxidant properties through OS modulation. This systemic review of 33 studies has documented its antioxidant activities. At the molecular and cellular levels, OS is a key in diabetes-induced DCM [18]. The antioxidant effects of *G. lucidum* are facilitated by increasing the antioxidant enzymes and inhibiting the enzymes involved in OS [33–35, 38]. *G. lucidum* consistently shows free radical scavenging activity against several free radicals including DPPH^·^, ABTS^·^+, HO^·^, and H_2_O_2_. As confirmed by the *in vitro* (chemical and cell-based) antioxidant tests, *G. lucidum* inhibits lipid peroxidation and protects against DNA damage.

*G. lucidum* modulates several signal pathways including Erk1/2, NF-*κ*B, and Wnt. Its antioxidant activity protects against inflammation and directly modulates immunity through scavenging radicals and through the oxidative signal pathways, thereby protecting the cells. These effects of *G. lucidum* may contribute to its positive influence on DCM.

DM is a state of persistent inflammation that upregulates mTOR at different levels of the myocardium, thereby influencing several signal pathways. The elevation of cellular cAMP levels disrupts phosphodiesterase-Rheb interaction, increases Rheb-mTOR interaction, and consequently leads to mTOR1 activation. Phosphodiesterase binds with Rheb and thereby inhibits the latter's ability to activate mTOR [71]. Heart myocardium responds to high blood glucose by adapting its energy metabolism and using only fatty acids as a substrate, thus increasing OS through the upregulation of NADPH-oxidases, NO synthases [72], and reversible oxidative modifications for myocardial titin elastic protein [73]. mTOR upregulation and oxidative modification alter titin-based stiffness and titin isoform composition, thereby impairing myocardium contractility. The PI3K-Akt-mTOR kinase axis regulates the composition of titin isoform [73]. OS decreases NO levels, leading to the impairment of the NO-soluble guanylate cyclase- (sGC-) cyclic guanosine monophosphate- (cGMP-) protein kinase G (PKG) pathway, an important regulator of cardiac contractility [72]. Chronic intrude accumulation to high free fatty acids downregulates PPAR-*α* and impairs mTOR-PPAR-*α*, thereby causing mitochondrial dysfunction in rodent cardiomyocytes and further deteriorating cardiac function through the inhibition of fatty acid oxidation and increase in intracellular fat accumulation. PPAR-*α* is involved in the upregulation of carnitine palmitoyltransferase I, which increases the uptake of long-chain fatty acid in the mitochondria and facilitates the beta-oxidation of fatty acids. mTOR-PPAR-*α* axis modification can lead to inflammation [74] and immune dysfunction [75]. mTOR upregulation leads to the impaired response to adrenergic stimulation in DCM mice and further reduces heart contractility [58]. mTOR inhibition improves contractility via the chronic administration of PDE inhibitor in animals and patients with diabetes [76] and restores the impaired response to adrenergic stimulation in DCM mice [58]. *G. lucidum* shows its effects via several signal pathways such as Wnt, Erk1/2, and NF-*κ*B pathway and consequently reduces the upregulated mTOR and its effects. mTOR is the main target of *G. lucidum*, and this finding supports its antioxidant and cardioprotective effects. *G. lucidum* inhibits the Wnt pathway [54] and may decrease the activity of mTOR via the Wnt/GSk/mTOR signal pathway. A pathologically stressed heart reactivates the Wnt signal pathway, which is modulated during left ventricular remodeling [77]. In heart cells, the Wnt pathway plays a role in the release of intracellular Ca^2+^ whose accumulation activates several Ca^+2^-sensitive proteins, fat and glucose metabolism, and cell fate decisions, such as renewal, differentiation, and apoptosis. Wnt dysregulation has an important role in cardiac diseases such as hypertrophy and fibrosis [78]. The Wnt pathway is important in the response to heart injuries leading to adverse effects on the heart [79] and is integrated with bioenergetic status to control mTOR activity [80]. Wnt is activated in late-stage inflammation of heart tissue [81]. *G. lucidum* suppresses Erk1/2 signaling [55] and consequently reduces the mTOR level. Erk1/2 signaling inhibits the TSC1/2 complex, which is the downregulator of mTOR, and thus activates mTOR [82]. The antioxidant properties of *G. lucidum* abolish the activation of the Erk pathway by OS. NADPH oxidase 2 is involved in Erk activation [83], and the inhibition of Erk/mTOR by *G. lucidum* also prevents NF-*κ*B. mTOR activates NF-*κ*B by phosphorylating the NF-*κ*B p65 subunit, increasing p65 nuclear translocation, and activating gene transcription. With its anti-inflammatory effect, *G. lucidum* inhibits NF-*κ*B via decreasing inflammatory mediators and cytokines such as TNF or IL-1, and innate immune response effectors activate NF-*κ*B via the IKK complex through I*κ*B protein phosphorylation with subsequent ubiquitination and degradation [84]. Inhibiting mTOR and NF-*κ*B may improve the contractility of the heart, abolish the angiotensin II-induced hypertrophic response of cardiomyocytes [83], and prevent heart failure. A prolonged NF-*κ*B activation promotes heart failure by evoking signals that induce chronic inflammation through the enhancement of cytokines including tumor necrosis factor, IL-1, and IL-6, commencing to endoplasmic reticulum stress responses and cell death [85].

**Figure 4:**
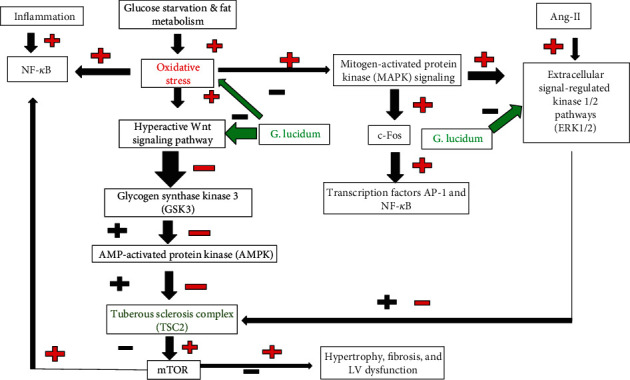
*G. lucidum* inhibits mTOR via several signal pathways (- inhibit; + stimulate). Red +/- effects of inflammation, glucose starvation and fat metabolism, and oxidative stress on different effectors of different pathways in cardiocytes; black +/- protective effects of *G lucidum* at different effectors of different pathways in cardiocytes.

Our results concluded that the antioxidant properties of *G. lucidum* and the cardioprotection of its polysaccharides may have a direct effect. Its free radical scavenging ability reduces OS and upregulates mTOR via several pathways including Wnt, Erk1/2, and NF-*κ*B/IKK/TOR, thereby improving myocardium contractility (Figure 4). The anti-inflammatory properties may enhance the cAMP/cGMP/mTOR/PPAR pathway and its related protein or/and pathway and mitochondrial function, thus improving myocardium hemostasis. Further study is needed to identify the specific target of GLP in heart tissues.

## Figures and Tables

**Table 1:** Association sore of 155 targets associated with diabetic cardiomyopathy in heart tissue.

	Target name	Target symbol	Association score		
			Data types Known drug	Data types Literature	Overall
1	Tripartite motif containing 55	TRIM55	0	0.177	0.177
2	Peroxisome proliferator-activated receptor alpha	PPARA	0	0.117	0.117
3	Mechanistic target of rapamycin kinase	MTOR	0.1	0.054	0.113
4	Interleukin 6	IL-6	0	0.113	0.113
5	Carnitine palmitoyltransferase 1B	CPT1B	0.1	0.000	0.100
6	Carnitine palmitoyltransferase 2	CPT2	0.1	0.000	0.100
7	Tripartite motif containing 54	TRIM54	0	0.081	0.081
8	Nuclear factor, erythroid 2 like 2	NFE2L2	0	0.072	0.072
9	Hydroxysteroid 11-beta dehydrogenase 1	HSD11B1	0	0.070	0.070
10	Fibroblast growth factor 1	FGF1	0	0.070	0.070
11	Colony-stimulating factor 3	CSF3	0	0.062	0.062
12	Beclin 1	BECN1	0	0.062	0.062
13	Cytochrome P450 family 2 subfamily J member 2	CYP2J2	0	0.061	0.061
14	Angiotensin I-converting enzyme 2	ACE2	0	0.060	0.060
15	Aldehyde dehydrogenase 2 family member	ALDH2	0	0.059	0.059
16	Glycogen synthase kinase 3 beta	GSK3B	0	0.057	0.057
17	Gelsolin	GSN	0	0.055	0.055
18	Toll-like receptor 2	TLR2	0	0.054	0.054
19	Parkin RBR E3 ubiquitin protein ligase	PRKN	0	0.054	0.054
20	Apelin	APLN	0	0.053	0.053
21	ST3 beta-galactoside alpha-2,3-sialyltransferase 4	ST3GAL4	0	0.052	0.052
22	Peroxisome proliferator activated receptor gamma	PPARG	0	0.052	0.052
23	Corin, serine peptidase	CORIN	0	0.052	0.052
24	Titin	TTN	0	0.049	0.049
25	Angiogenin	ANG	0	0.049	0.049
26	Protein kinase D1	PRKD1	0	0.049	0.049
27	PPARG coactivator 1 alpha	PPARGC1A	0	0.048	0.048
28	Vascular endothelial growth factor A	VEGFA	0	0.048	0.048
29	Insulin-like growth factor 1	IGF1	0	0.047	0.047
30	CD36 molecule	CD36	0	0.047	0.047
31	Nitric oxide synthase 3	NOS3	0	0.046	0.046
32	Apolipoprotein A1	APOA1	0	0.044	0.044
33	Gap junction protein alpha 1	GJA1	0	0.041	0.041
34	Calsequestrin 2	CASQ2	0	0.041	0.041
35	Decorin	DCN	0	0.040	0.040
36	Urocortin	UCN	0	0.040	0.040
37	Cellular communication network factor 2	CCN2	0	0.040	0.040
38	Matrix metallopeptidase 2	MMP2	0	0.040	0.040
39	Periostin	POSTN	0	0.039	0.039
40	Fibroblast growth factor 2	FGF2	0	0.039	0.039
41	BCL6 transcription repressor	BCL6	0	0.039	0.039
42	Tax1-binding protein 1	TAX1BP1	0	0.038	0.038
43	Solute carrier family 2 member 4	SLC2A4	0	0.038	0.038
44	Rho-associated coiled-coil containing protein kinase 2	ROCK2	0	0.037	0.037
45	NADPH oxidase 4	NOX4	0	0.036	0.036
46	Mitogen-activated protein kinase 9	MAPK9	0	0.036	0.036
47	Insulin-like growth factor 2	IGF2	0	0.036	0.036
48	Angiotensin II receptor type 2	AGTR2	0	0.036	0.036
49	Lipoprotein lipase	LPL	0	0.036	0.036
50	Insulin receptor	INSR	0	0.035	0.035
51	Angiopoietin 1	ANGPT1	0	0.035	0.035
52	Interleukin 33	IL33	0	0.035	0.035
53	Caveolin 3	CAV3	0	0.034	0.034
54	Angiotensin I-converting enzyme	ACE	0	0.034	0.034
55	Patatin-like phospholipase domain containing 2	PNPLA2	0	0.034	0.034
56	ATPase sarcoplasmic/endoplasmic reticulum Ca^2+^ transporting 2	ATP2A2	0	0.033	0.033
57	Glucokinase	GCK	0	0.032	0.032
58	Dimethylarginine dimethylaminohydrolase 2	DDAH2	0	0.032	0.032
59	Xenotropic and polytropic retrovirus receptor 1	XPR1	0	0.032	0.032
60	Vascular endothelial growth factor B	VEGFB	0	0.032	0.032
61	Phosphodiesterase 5A	PDE5A	0	0.031	0.031
62	MAPK-activated protein kinase 2	MAPKAPK2	0	0.031	0.031
63	Heat shock protein family E (Hsp10) member 1	HSPE1	0	0.031	0.031
64	Sirtuin 2	SIRT2	0	0.031	0.031
65	DIRAS family GTPase 3	DIRAS3	0	0.030	0.030
66	SMAD family member 3	SMAD3	0	0.030	0.030
67	Dual specificity phosphatase 5	DUSP5	0	0.030	0.030
68	Kruppel-like factor 4	KLF4	0	0.030	0.030
69	Ryanodine receptor 2	RYR2	0	0.029	0.029
70	Prohibitin	PHB	0	0.029	0.029
71	Estrogen related receptor gamma	ESRRG	0	0.028	0.028
72	Nebulin	NEB	0	0.028	0.028
73	Peroxiredoxin 3	PRDX3	0	0.028	0.028
74	Adrenoceptor beta 2	ADRB2	0	0.028	0.028
75	Solute carrier family 9 member A1	SLC9A1	0	0.028	0.028
76	Transglutaminase 2	TGM2	0	0.027	0.027
77	Poly(ADP-ribose) polymerase 1	PARP1	0	0.027	0.027
78	Insulin receptor substrate 1	IRS1	0	0.027	0.027
79	Voltage dependent anion channel 1	VDAC1	0	0.026	0.026
80	AKT serine/threonine kinase 1	AKT1	0	0.025	0.025
81	Myocyte enhancer factor 2A	MEF2A	0	0.025	0.025
82	Dual specificity phosphatase 1	DUSP1	0	0.025	0.025
83	Musculin	MSC	0	0.025	0.025
84	Diacylglycerol kinase zeta	DGKZ	0	0.024	0.024
85	Death associated protein kinase 2	DAPK2	0	0.024	0.024
86	Solute carrier family 25 member 4	SLC25A4	0	0.023	0.023
87	SMAD family member 7	SMAD7	0	0.023	0.023
88	Natriuretic peptide A	NPPA	0	0.023	0.023
89	Coiled-coil domain containing 47	CCDC47	0	0.022	0.022
90	Lipase E, hormone sensitive type	LIPE	0	0.022	0.022
91	Leptin	LEP	0	0.022	0.022
92	Arylsulfatase A	ARSA	0	0.021	0.021
93	Nitric oxide synthase 2	NOS2	0	0.021	0.021
94	Nuclear receptor subfamily 3 group C member 2	NR3C2	0	0.021	0.021
95	Sirtuin 3	SIRT3	0	0.021	0.021
96	Plasminogen	PLG	0	0.020	0.020
97	Spindlin 1	SPIN1	0	0.020	0.020
98	Serpin family E member 1	SERPINE1	0	0.020	0.020
99	Tachykinin receptor 1	TACR1	0	0.020	0.020
100	RNA binding fox-1 homolog 2	RBFOX2	0	0.020	0.020
101	Fatty acid binding protein 4	FABP4	0	0.019	0.019
102	Potassium voltage-gated channel subfamily H member 2	KCNH2	0	0.019	0.019
103	Cell adhesion molecule 1	CADM1	0	0.019	0.019
104	Prolylcarboxypeptidase	PRCP	0	0.018	0.018
105	Nucleotide-binding oligomerization domain containing 1	NOD1	0	0.018	0.018
106	Activating transcription factor 3	ATF3	0	0.018	0.018
107	Vasoactive intestinal peptide	VIP	0	0.018	0.018
108	Egl-9 family hypoxia inducible factor 3	EGLN3	0	0.018	0.018
109	Fibronectin 1	FN1	0	0.018	0.018
110	Endothelin 1	EDN1	0	0.018	0.018
111	C-C motif chemokine ligand 2	CCL2	0	0.018	0.018
112	Solute carrier family 5 member 1	SLC5A1	0	0.018	0.018
113	Fibrinogen-like 2	FGL2	0	0.017	0.017
114	Monoamine oxidase A	MAOA	0	0.017	0.017
115	Sphingosine-1-phosphate receptor 1	S1PR1	0	0.017	0.017
116	Signal transducer and activator of transcription 3	STAT3	0	0.017	0.017
117	Toll-like receptor 3	TLR3	0	0.017	0.017
118	Tripartite motif containing 63	TRIM63	0	0.017	0.017
119	TIMP metallopeptidase inhibitor 2	TIMP2	0	0.017	0.017
120	Nerve growth factor	NGF	0	0.017	0.017
121	Natriuretic peptide receptor 2	NPR2	0	0.016	0.016
122	Cyclin-dependent kinase inhibitor 1A	CDKN1A	0	0.016	0.016
123	Cathepsin D	CTSD	0	0.016	0.016
124	Thrombospondin 1	THBS1	0	0.015	0.015
125	Kinase insert domain receptor	KDR	0	0.015	0.015
126	Serine/threonine kinase 11	STK11	0	0.015	0.015
127	Enolase 3	ENO3	0	0.015	0.015
128	Gasdermin D	GSDMD	0	0.015	0.015
129	Cytochrome c, somatic	CYCS	0	0.015	0.015
130	Kallikrein B1	KLKB1	0	0.015	0.015
131	TIMP metallopeptidase inhibitor 4	TIMP4	0	0.015	0.015
132	Transforming growth factor beta 3	TGFB3	0	0.015	0.015
133	Zinc finger and BTB domain containing 16	ZBTB16	0	0.015	0.015
134	Collagen type I alpha 1 chain	COL1A1	0	0.015	0.015
135	Endothelin receptor type A	EDNRA	0	0.014	0.014
136	Cellular communication network factor 1	CCN1	0	0.014	0.014
137	Secreted protein acidic and cysteine rich	SPARC	0	0.014	0.014
138	Glucagon like peptide 1 receptor	GLP1R	0	0.014	0.014
139	Cystatin C	CST3	0	0.014	0.014
140	Intercellular adhesion molecule 1	ICAM1	0	0.014	0.014
141	Elastin	ELN	0	0.014	0.014
142	Tenascin C	TNC	0	0.014	0.014
143	PTEN-induced kinase 1	PINK1	0	0.014	0.014
144	Calpastatin	CAST	0	0.014	0.014
145	CCAAT enhancer binding protein beta	CEBPB	0	0.012	0.012
146	Acyl-coA thioesterase 1	ACOT1	0	0.012	0.012
147	G protein-coupled bile acid receptor 1	GPBAR1	0	0.010	0.010
148	Annexin A1	ANXA1	0	0.010	0.010
149	Apolipoprotein L2	APOL2	0	0.008	0.008
150	Natriuretic peptide B	NPPB	0	0.008	0.008
151	Leptin receptor	LEPR	0	0.008	0.008
152	Serum response factor	SRF	0	0.008	0.008
153	Heat shock protein family B (small) member 3	HSPB3	0	0.007	0.007
154	Angiotensin II receptor type 1	AGTR1	0	0.007	0.007
155	Protein phosphatase 5 catalytic subunit	PPP5C	0	0.007	0.007

**Table 2:** Nineteen pathway types involved in the heart tissues of 155 targets expressed.

No.	Pathway (No. of targets)
1.	Signal transduction (63)
2.	Immune system (47)
3.	Metabolism of proteins (39)
4.	Metabolism (31)
5.	Gene expression (transcription) (25)
6.	Hemostasis (23)
7.	Disease (22)
8.	Developmental biology (20)
9.	Extracellular matrix organization (18)
10.	Cellular responses to external stimuli (14)
11.	Transport of small molecules (11)
12.	Muscle contraction (11)
13.	Vesicle-mediated transport (10)
14.	Organelle biogenesis and maintenance (4)
15.	Programmed cell death (4)
16.	Autophagy (4)
17.	Neuronal system (3)
18.	Cell cycle (3)
19.	Circadian clock (3)

**Table 3:** mTOR score association with 49 heart diseases.

No.	Heart disease	Association score				
		Data types Genetic	Data types Known drug	Data types Literature	Data types Animal model	Overall
1	Heart disease	0.00041	0.79550	0.14161	0.19028	0.8588
2	Cardiomyopathy	0.00000	0.77847	0.11930	0.19028	0.8393
3	Hypertrophic cardiomyopathy	0.00000	0.77222	0.10214	0.00000	0.7978
4	Heart failure	0.00000	0.25000	0.05235	0.00000	0.2631
5	Dilated cardiomyopathy	0.00000	0.00000	0.07568	0.19028	0.2092
6	Congestive heart failure	0.00000	0.20000	0.02636	0.00000	0.2066
7	Diastolic heart failure	0.00000	0.20000	0.00000	0.00000	0.2000
8	Barth syndrome	0.00000	0.00000	0.00000	0.19028	0.1903
9	Coronary heart disease	0.00000	0.00000	0.12009	0.00000	0.1201
10	Diabetic cardiomyopathy	0.00000	0.10000	0.05391	0.00000	0.1135
11	Coronary artery disease	0.00000	0.00000	0.10961	0.00000	0.1096
12	Systemic scleroderma	0.00000	0.00000	0.09914	0.00000	0.0991
13	Cardiotoxicity	0.00000	0.00000	0.09016	0.00000	0.0902
14	Glycogen storage disease due to acid maltase deficiency	0.00000	0.00000	0.08380	0.00000	0.0838
15	Myocardial infarction	0.00000	0.00000	0.06467	0.00000	0.0647
16	Persistent truncus arteriosus	0.00000	0.00000	0.06144	0.00000	0.0614
17	Heart neoplasm	0.00000	0.00000	0.06126	0.00000	0.0613
18	Emery-Dreifuss muscular dystrophy	0.00000	0.00000	0.05780	0.00000	0.0578
19	Ischemia reperfusion injury	0.00000	0.00000	0.05702	0.00000	0.0570
20	Myocardial ischemia	0.00000	0.00000	0.05658	0.00000	0.0566
21	Carney complex	0.00000	0.00000	0.05494	0.00000	0.0549
22	Down syndrome	0.00000	0.00000	0.05488	0.00000	0.0549
23	Cardiac rhabdomyoma	0.00000	0.00000	0.05475	0.00000	0.0547
24	Autosomal dominant Emery-Dreifuss muscular dystrophy	0.00000	0.00000	0.05280	0.00000	0.0528
25	Polyarteritis nodosa	0.00000	0.00000	0.04343	0.00000	0.0434
26	Steinert myotonic dystrophy	0.00000	0.00000	0.04273	0.00000	0.0427
27	Acute myocardial infarction	0.00000	0.00000	0.03798	0.00000	0.0380
28	Cardiac arrhythmia	0.00041	0.00000	0.03721	0.00000	0.0373
29	Myocarditis	0.00000	0.00000	0.03263	0.00000	0.0326
30	Duchenne muscular dystrophy	0.00000	0.00000	0.03253	0.00000	0.0325
31	Gaucher disease	0.00000	0.00000	0.03230	0.00000	0.0323
32	Cardiac arrest	0.00000	0.00000	0.02847	0.00000	0.0285
33	Atrial fibrillation	0.00000	0.00000	0.02720	0.00000	0.0272
34	Aortic stenosis	0.00000	0.00000	0.01910	0.00000	0.0191
35	Acute coronary syndrome	0.00000	0.00000	0.01900	0.00000	0.0190
36	Sleep disorder	0.00000	0.00000	0.01840	0.00000	0.0184
37	Williams syndrome	0.00000	0.00000	0.01640	0.00000	0.0164
38	Supravalvular aortic stenosis	0.00000	0.00000	0.01640	0.00000	0.0164
39	Autoimmune myocarditis	0.00000	0.00000	0.01560	0.00000	0.0156
40	Friedreich ataxia	0.00000	0.00000	0.01480	0.00000	0.0148
41	Obstructive sleep apnea	0.00000	0.00000	0.01480	0.00000	0.0148
42	PHACE syndrome	0.00000	0.00000	0.01440	0.00000	0.0144
43	Glycogen storage disease due to LAMP-2 deficiency	0.00000	0.00000	0.01440	0.00000	0.0144
44	Idiopathic pulmonary arterial hypertension	0.00000	0.00000	0.01400	0.00000	0.0140
45	Fabry disease	0.00000	0.00000	0.01340	0.00000	0.0134
46	Becker muscular dystrophy	0.00000	0.00000	0.00840	0.00000	0.0084
47	Hemopericardium	0.00000	0.00000	0.00720	0.00000	0.0072
48	Aortic coarctation	0.00000	0.00000	0.00680	0.00000	0.0068
49	Atrial flutter	0.00041	0.00000	0.00000	0.00000	0.0004

**Table 4:** *In vivo* studies of *G. lucidum.*

No.	Animal	Form	Dosage (mg/kg)	Antioxidant parameters	Biological activity	Pathway	References
1	CCl_4_-induced acute liver injury mice	GLPS	100 - 150	NOS CYP2E1 MDA, GSH	Suppressing free radical lipid peroxidation	Decreasing of the protein expression levels of NLRP3, ASC, and caspase-1 in acute liver injury. ASC (apoptosis-associated speck-like protein) NLRP3 (NOD-like receptor 3) Caspase-1 GAPDH (glyceraldehyde-3-phosphate hydrogenase	[22]

2	Croton oil applied skin edema in rats	Ethanol extract of sporocarps	500 and 1000 mg/kg		Antiperoxidative, anti-inflammatory, and antimutagenic activities	Direct anti-inflammatory and free radical scavenging properties of the extract	[23]

3	Photoreceptor cell lesions induced by N-methyl-N-nitrosourea (MNU) in female SD arts	Ganoderma spore lipid (GSL)	500, 1000, 2000, and 4000 mg/kg	Expressions of Bax, Bcl-xl, and caspase-3	Improve A-wave amplitude (*μ*v) decreased apoptosis levels	Regulate the expressions of Bax, Bcl-xl, and caspases-3, inhibiting MNU-induced rat, photoreceptor cell apoptosis, and protecting retinal function	[30]

4	A carotid-artery-ligation mouse model	Ganoderma triterpenoid (GT)	300 mg/kg/day	Intimal hyperplasia structural changes VCAM-1, TNF-*α*, and IL-6	Atheroprotective properties	Endothelin-1, von Willebrand factor, and monocyte chemoattractant protein-1	[33]

5	Swimming-induced oxidative stress in skeletal muscle mice	GLPS	50, 100, and 200 mg/kg	SOD, GPX, and CAT activities as well as by the MDA levels	Attenuates exercise-induced oxidative stress in skeletal muscle	Increasing antioxidant enzyme activities and decrease the MDA levels. Protective effects against exhaustive exercise-induced oxidative stress	[32]

6	Rat gastric cancer model	GLPS	400-800 mg/kg for 20 weeks	SOD, CAT, and GSH-Px	Antioxidant	Induced the levels of serum IL-6 and TNF-*α* levels and increased the levels of serum IL-2, IL-4, and IL-10 in GLP-treated rats compared to gastric cancer model rats	[37]

7	BALB/c female mice	GLPS i.p. daily	50 mg/kg, 100 mg/kg, and 200 mg/kg	SOD and GSH-Px	Antioxidant	Improved immunity in mice. Increased thymus and spleen index; improved SOD and GSH-Px contents in the mice body	[31]

8	T2DM rats	GLPS	200, 400, and 800 mg × kg^−1^ for 16 weeks	NO, SOD, MDA, GSH-Px, and CAT MDA in cardiac tissue	Antioxidation in cardiac tissue of T2DM rats	Reduce MDA in cardiac tissue and improve the myocardial ultrastructure	[34]

9	Male BALB/c mice (age19-21 months) (aged mice)	Ethanolic extract of *G*. *lucidum*	50 and 250 mg/kg, once daily for 15 days	GSH Mn-SOD, GPx, and GST	Antioxidant in heart tissues	Elevated the levels of GSH as well as activities of MnSOD, GPx, and GST and decreased significantly the levels of lipid peroxidation, AOPP, and ROS. Improve the age-related decline of antioxidant status which was partly ascribed to free radical scavenging activity	[38]

10	B16 mouse melanoma	Methanol extract containing total terpenoids (GLme) and a purified methanol extract containing mainly acidic terpenoids (GLpme)	A daily i.p. injection of 100 mg/kg body weight (b.w.)	Production of oxygen radical caspase-dependent apoptotic cell death-mediated production of reactive oxygen species	Anticancer	The mechanism of antitumor activity of GLme comprised inhibition of cell proliferation and induction of caspase-dependent apoptotic cell death mediated by upregulated p53 and inhibited Bcl-2 expression	[86]

11	With non-insulin-dependent diabetes mellitus (NIDDM)	Ganoderma *lucidum* spores	250 mg/kg × d, for 10	Xanthine oxidase (XOD), myeloperoxidase (MPO), and mitochondrial succinate dehydrogenase (SDH) in the testis	Reducing free radical-induceddamage to the testicular tissue	Protect the testis of diabetic rats by reducing free radical-induced damage to the testicular tissue and enhancing the activity of SDH	[35]

12	Epididymal cells of type 2 diabetes rats	*Ganoderma lucidum* spores (GLS)	250 mg/kg × d, for 10 weeks	Contents of mitochondrial calcium & cytochrome C	Antipoptosis induced by DM	Protect epididymal cells and counteract their apoptosis in diabetic condition	[36]

13	Liver tissue of rats	*Ganoderma lucidum* peptide	27.1 *μ*g/mL	Malondialdehyde level	Antioxidant	Substantial antioxidant activity in the rat liver tissue homogenates and mitochondrial membrane peroxidation systems	[87]

14	Lupus mice	Ganoderma tsugae	0.5 mg/kg/day	Decreased proteinuria, decreased serum levels of antidsDNA autoantibody	Prevention of autoantibody	Prevention of autoantibody formation	[88]

**Table 5:** *In vitro* studies of *G. lucidum.*

No.	Form	Conc.	Chemical antioxidant tests	Biological text of in vitro	Exp. parameters	Biological activity	Pathway	References
1	GLP	0.5-3.0 mg/mL	RS FR	=	Scavenging of free radicals and reducing power	Antioxidant	NM	[89]
2	*G*. *lucidum* and Egyptian Chlorella vulgaris	CVE (63.5 *μ*g/mL) was mixed with GLE (4.1 *μ*g/mL)	RS FR AP Other tests	Lipopolysaccharide-stimulated white blood cells	Nitric oxide, tumor necrosis factor- (TNF-) *α*	Antioxidant and anti-inflammatory	Downregulate NF-*κ*B	[39]
3	Polysaccharides in *G*. *lucidum*	2 mg/mL	RS FR AP Other tests	NM	Radical scavenging reducing power	Antioxidant	NM	[90]
4	*G*. *lucidum* extract	50 mg	RS FR AP Other tests	NM	Reducing power	Antimicrobial and antioxidant	NM	[40]
5	*Ganoderma lucidum* G2	0.32 mg	RS FR AP Other tests	DNA protection	Radical scavenging reducing power	Antimicrobial and antioxidant	NM	[41]
6	Protein extracts	2–13 *μ*g protein/mL	AP Other tests	DNA protection	Radical scavenging reducing power	Antioxidant, antibacterial	NM	[42]
7	Polysaccharides extraction	=	FR AP Other tests	MCF-7 breast cancer cell line and HeLa cells	Radical scavenging	Antioxidant Anticancer	NM	[43]
8	*G*. *lucidum* and G. resinaceum	0.1–1 & 0.64 ± 0.04 0–2.25 mg/mL	FR AP Other tests	In vitro cell line	Radical-scavenging chelating lipoxygenase assay	Antiproliferative & antioxidant	NM	[44]
9	Diff, organic solvent o *G*. *lucidum*	1-200 *μ*g/mL	FR AP Other tests	NM	Radical scavenging, chelating lipid peroxidation	Antioxidant Anticholinesterase	NM	[45]
10	Both aqueous and methanolic extracts	0.2–30 mg/mL of extraction	FR AP Other tests	NM	Radical scavenging, chelating lipid peroxidation	Antioxidant	NM	[46]
11	Low-molecular-weight *β*-1,3-glucan	0–200 *μ*g/mL	AP Other tests	Mouse monocyte-macrophage cell line, RAW 264.7	H_2_O_2_-induced apoptosis	Antioxidant	Attenuating intracellular reactive oxygen species (ROS) and inhibiting sphingomyelinase (SMase) activity	[51]
12	Polysaccharides	0.16-10 mg/mL	FR AP Other tests	NM	Radical scavenging, chelating reducing power	Antioxidant	NM	[47]
13	*G*. *lucidum*water-soluble and water-insoluble	80-1100 *μ*g/ml	FR AP Other tests	Human uroepithelial cell (HUC-PC) cells	Radical scavenging, chelating reducing power	Antioxidant	Oxidative DNA damage. Lingzhi-induced apoptosis in bladder chemoprevention	[48]
14	*Ganoderma lucidum* polysaccharides	0.1-0.6 mg/ml	RS	CCl-induced injury hepatocytes DNA protection	MDA, SOD, CYP3A, caspase-3, andcaspase-8	Suppressing inflammatory responses	Reduction of NF-*κ*B activation inhibition of caspase-3, caspase-6, and caspase-9, indicating and suppression extrinsic-induced apoptosis	[52]
15	Ganoderic acid A	10-80 lM/mL	NM	Pancreatic cells	Radical scavenging Antiproliferative	Antioxidant Anticancer	*β*-Catenin in Wnt signaling pathway	[54]
16	Aqueous extract of *G*. *lucidum*	5-20 *μ*L	NM	DNA protection	Radical scavenging	Antioxidant DNA repair	Enhancing reactivity of apurinic/apyrimidinic endonucleases (APE1) a major enzyme of base excision repair (BER)	[91]
17	Methanolic extract of *G*. *lucidum*	65 & 130 *μ*g/mL	NM	Human gastric tumor cells	Increased the formation of autophagosomes	Induces autophagy	Increasing of the cellular levels of LC3-II and decreasing p62 (autophagy-related protein)	[92]
18	*G*. *lucidum* (GLPS) and G. sinense (GSPS)	19–300 *μ*g/mL	NM	RAW 264.7 mouse macrophage cells	Nitric oxide secretion of cytokines	Immunomodulatory	Promoting macrophage phagocytosis, increasing their release of nitric oxide and cytokines interleukin- (IL-) 1a, IL-6, IL-10, and tumor necrosis factor-*α*	[56]
19	Proteopolysaccharide from *G*. *lucidum*	2 - 10 *μ*g/mL	NM	RAW264.7, a mouse macrophage cell line	Nitrite production Expression levels of cytokines	Activation the immune system by modulating cytokine production.	NM	[57]

NM = not mention; RS = radical scavenging; FR = ferric reducing; AP = antilipid peroxidation.

## Abbreviations

ABTS^·^+: 2,2′-azino-bis (3-ethylbenzthiazoline-6-sulphonic acid) radical
AOPP: Advanced oxidation protein products
Bax: BCL2 associated X, apoptosis regulator
Bcl-xl: B-cell lymphoma-extra large
BECN1: Beclin 1
cAMP: Cyclic adenosine monophosphate
CAT: Catalase
CCl_4_: Carbon tetrachloride
c-Fos: A protooncogene
cGMP: Cyclic guanosine monophosphate
CPT1B and CPT2: Carnitine palmitoyltransferase 1B and 2
CYP2E1: Cytochrome P450 2E1
DCM: Diabetic cardiomyopathy
DM: Diabetic mellitus
DNA: Deoxyribonucleic acid
DPPH^·^: 2,2-diphenylpicrylhydrazyl radical
EMBL: European Molecular Biology Laboratory
Erk1/2: Extracellular signal-regulated kinase
GLPs: *Ganoderma lucidum* polysaccharides
GPx: Glutathione peroxidase
GR: Glutathione reductase
GSH: Reduced glutathione
GSH-Px: Glutathione peroxidase
GST: Glutathione-S-transferase
GTs: Ganoderma triterpenoids
H_2_O_2_: Hydrogen peroxide radicals
HO^·^: Hydroxyl radical
IL-6: Interleukin 6
LC3: Light chain 3
LPS: Lipopolysaccharide
MAF1: Protein negative regulator of RNA polymerase III
MCF-7 cells: Breast cancer cell line
MDA: Malondialdehyde level
Mn-SOD: Manganese-superoxide dismutase
MNU: N-methyl-N-nitrosourea
MPO: Myeloperoxidase
mTOR: Mammalian target of rapamycin
mTORC: mTOR complex
NF-*κ*B: Nuclear factor-*κ*B
NO: Nitrous oxide
NOS: Nitric oxide synthase
OS: Oxidative stress
OTP: Open Targets Platform
PDE: Phosphodiesterase
PKG: Protein kinase G
PML: Promyelocytic leukemia.

## Supplementary Materials

Supplementary Materials Supplementary Table 1: association sore of 309 targets associated with diabetic cardiomyopathy in 30 recorded tissues. Supplementary Table 2: thirty-two tissue organs expressed 309 targets and types of pathways.

## References

[1] Raghavan S., Vassy J. L., Ho Y. L. (2019). Diabetes mellitus-related all-cause and cardiovascular mortality in a national cohort of adults. *Journal of the American Heart Association*.

[2] Glovaci D., Fan W., Wong N. D. (2019). Epidemiology of diabetes mellitus and cardiovascular disease. *Current Cardiology Reports*.

[3] Bertoluci M. C., Rocha V. Z. (2017). Cardiovascular risk assessment in patients with diabetes. *Diabetology & Metabolic Syndrome*.

[4] Williams L. J., Nye B. G., Wende A. R. (2017). Diabetes-related cardiac dysfunction. *Endocrinology and Metabolism*.

[5] Karuranga S., da Rocha Fernandes J., Huang Y., Malanda B. (2017). *IDF Diabetes Atlas*.

[6] Battiprolu P. K., Gillette T. G., Wang Z. V., Lavandero S., Hill J. A. (2010). Diabetic cardiomyopathy: mechanisms and therapeutic targets. *Drug Discovery Today: Disease Mechanisms*.

[7] Booth G. L., Kapral M. K., Fung K., Tu J. V. (2006). Relation between age and cardiovascular disease in men and women with diabetes compared with non-diabetic people: a population-based retrospective cohort study. *The Lancet*.

[8] Wannamethee S. G., Shaper A. G., Whincup P. H., Lennon L., Sattar N. (2011). Impact of diabetes on cardiovascular disease risk and all-cause mortality in older men: influence of age at onset, diabetes duration, and established and novel risk factors. *Archives of Internal Medicine*.

[9] Boudina S., Abel E. D. (2007). Diabetic cardiomyopathy revisited. *Circulation*.

[10] Mandavia C. H., Aroor A. R., DeMarco V. G., Sowers J. R. (2013). Molecular and metabolic mechanisms of cardiac dysfunction in diabetes. *Life Sciences*.

[11] Poirier P., Bogaty P., Garneau C., Marois L., Dumesnil J. G. (2001). Diastolic dysfunction in normotensive men with well-controlled type 2 diabetes: importance of maneuvers in echocardiographic screening for preclinical diabetic cardiomyopathy. *Diabetes Care*.

[12] Ernande L., Bergerot C., Rietzschel E. R. (2011). Diastolic dysfunction in patients with type 2 diabetes mellitus: is it really the first marker of diabetic cardiomyopathy?. *Journal of the American Society of Echocardiography*.

[13] Xi S., Zhou G., Zhang X., Zhang W., Cai L., Zhao C. (2009). Protective effect of total aralosides of Aralia elata (Miq) Seem (TASAES) against diabetic cardiomyopathy in rats during the early stage, and possible mechanisms. *Experimental & Molecular Medicine*.

[14] Sun X., Chen R. C., Yang Z. H. (2014). Taxifolin prevents diabetic cardiomyopathy in vivo and in vitro by inhibition of oxidative stress and cell apoptosis. *Food and Chemical Toxicology*.

[15] Zhou G., Li C., Cai L. (2004). Advanced glycation end-products induce connective tissue growth factor-mediated renal fibrosis predominantly through transforming growth factor *β*-independent pathway. *The American Journal of Pathology*.

[16] Pereira L., Matthes J., Schuster I. (2006). Mechanisms of [Ca2+] i transient decrease in cardiomyopathy of db/db type 2 diabetic mice. *Diabetes*.

[17] Sulaiman M., Matta M. J., Sunderesan N. R., Gupta M. P., Periasamy M., Gupta M. (2010). Resveratrol, an activator of SIRT1, upregulates sarcoplasmic calcium ATPase and improves cardiac function in diabetic cardiomyopathy. *American Journal of Physiology-Heart and Circulatory Physiology*.

[18] Schaffer S. W., Jong C. J., Mozaffari M. (2012). Role of oxidative stress in diabetes-mediated vascular dysfunction: unifying hypothesis of diabetes revisited. *Vascular Pharmacology*.

[19] Wachtel-Galor S., Yuen J., Buswell J. A., Benzie I. F. (2011). Ganoderma lucidum (Lingzhi or Reishi). *Herbal Medicine: Biomolecular and Clinical Aspects*.

[20] Kwon O., Lee C.-S., Park Y.-J. (2019). SNP and SCAR markers for specific discrimination of antler-shaped Ganoderma lucidum. *Microorganisms*.

[21] Wang X.-C., Xi R.-J., Li Y., Wang D.-M., Yao Y.-J. (2012). The species identity of the widely cultivated Ganoderma,‘G. lucidum’(Ling-zhi), in China. *PLoS One*.

[22] Chen Y.-S., Chen Q. Z., Wang Z. J., Hua C. (2019). Anti-inflammatory and hepatoprotective effects of Ganoderma lucidum polysaccharides against carbon tetrachloride-induced liver injury in Kunming mice. *Pharmacology*.

[23] Lakshmi B., Ajith T. A., Sheena N., Gunapalan N., Janardhanan K. K. (2003). Antiperoxidative, anti-inflammatory, and antimutagenic activities of ethanol extract of the mycelium of Ganoderma lucidum occurring in South India. *Teratogenesis, Carcinogenesis, and Mutagenesis*.

[24] Koscielny G., An P., Carvalho-Silva D. (2017). Open Targets: a platform for therapeutic target identification and validation. *Nucleic Acids Research*.

[25] Carvalho-Silva D., Pierleoni A., Pignatelli M. (2019). Open Targets Platform: new developments and updates two years on. *Nucleic Acids Research*.

[26] Liberati A. (2009). The PRISMA statement for reporting systematic reviews and meta-analyses of studies that evaluate health care interventions: explanation and elaboration. *Annals of internal medicine*.

[27] Hsu P. P., Kang S. A., Rameseder J. (2011). The mTOR-regulated phosphoproteome reveals a mechanism of mTORC1-mediated inhibition of growth factor signaling. *Science*.

[28] Kim D.-H., Sarbassov D. D., Ali S. M. (2002). mTOR interacts with raptor to form a nutrient-sensitive complex that signals to the cell growth machinery. *Cell*.

[29] Brugarolas J., Lei K., Hurley R. L. (2004). Regulation of mTOR function in response to hypoxia by REDD1 and the TSC1/TSC2 tumor suppressor complex. *Genes & Development*.

[30] Gao Y., Deng X. G., Sun Q. N., Zhong Z. Q. (2010). Ganoderma spore lipid inhibits N-methyl-N-nitrosourea-induced retinal photoreceptor apoptosis in vivo. *Experimental Eye Research*.

[31] Wang J., Wang Y., Liu X., Yuan Y., Yue T. (2013). Free radical scavenging and immunomodulatory activities of Ganoderma lucidum polysaccharides derivatives. *Carbohydrate Polymers*.

[32] Zhonghui Z., Xiaowei Z., Fang F. (2014). Ganoderma lucidum polysaccharides supplementation attenuates exercise-induced oxidative stress in skeletal muscle of mice. *Saudi journal of biological sciences*.

[33] Hsu P.-L., Lin Y. C., Ni H., Mo F. E. (2018). Ganoderma triterpenoids exert antiatherogenic effects in mice by alleviating disturbed flow-induced oxidative stress and inflammation. *Oxidative Medicine and Cellular Longevity*.

[34] Xue H., Qiao J., Meng G. (2010). Effect of Ganoderma lucidum polysaccharides on hemodynamic and antioxidation in T2DM rats. *China journal of Chinese materia medica*.

[35] Wang S., Qin W. B., Kang Y. M. (2008). Intervention effect of ganoderma lucidum spores on the changes of XOD, MPO and SDH in the testis tissue of NIDDM rats. *National Journal of Andrology*.

[36] Ma X., Zhou C. F., Wang S. Q. (2007). Effects of ganoderma lucidum spores on mitochondrial calcium ion and cytochrome C in epididymal cells of type 2 diabetes rats. *National Journal of Andrology*.

[37] Pan K., Jiang Q., Liu G., Miao X., Zhong D. (2013). Optimization extraction of Ganoderma lucidum polysaccharides and its immunity and antioxidant activities. *International Journal of Biological Macromolecules*.

[38] Sudheesh N. P., Ajith T. A., Ramnath V., Janardhanan K. K. (2010). Therapeutic potential of _Ganoderma lucidum_ (Fr.) P. Karst. against the declined antioxidant status in the mitochondria of post-mitotic tissues of aged mice. *Clinical Nutrition*.

[39] Abu-Serie M. M., Habashy N. H., Attia W. E. (2018). In vitro evaluation of the synergistic antioxidant and anti-inflammatory activities of the combined extracts from Malaysian Ganoderma lucidum and Egyptian Chlorella vulgaris. *BMC Complementary and Alternative Medicine*.

[40] Sharif S., Shahid M., Mushtaq M., Akram S., Rashid A. (2017). Wild mushrooms: a potential source of nutritional and antioxidant attributes with acceptable toxicity. *Preventive nutrition and food science*.

[41] Sarnthima R., Khammaung S., Sa-ard P. (2017). Culture broth of Ganoderma lucidum exhibited antioxidant, antibacterial and *α*-amylase inhibitory activities. *Journal of Food Science and Technology*.

[42] Sa-ard P., Sarnthima R., Khammuang S., Kanchanarach W. (2015). Antioxidant, antibacterial and DNA protective activities of protein extracts from Ganoderma lucidum. *Journal of Food Science and Technology*.

[43] Chen P., Yong Y., Gu Y., Wang Z., Zhang S., Lu L. (2015). Comparison of antioxidant and antiproliferation activities of polysaccharides from eight species of medicinal mushrooms. *International journal of medicinal mushrooms*.

[44] Saltarelli R., Ceccaroli P., Buffalini M. (2015). Biochemical characterization and antioxidant and antiproliferative activities of different Ganoderma collections. *Journal of Molecular Microbiology and Biotechnology*.

[45] Tel G., Ozturk M., Duru M. E., Turkoglu A. (2014). Antioxidant and anticholinesterase activities of five wild mushroom species with total bioactive contents. *Pharmaceutical Biology*.

[46] Rani P., Lal M. R., Maheshwari U., Krishnan S. (2015). Antioxidant potential of lingzhi or reishi medicinal mushroom, Ganoderma lucidum (higher basidiomycetes) cultivated on Artocarpus heterophyllus sawdust substrate in India. *International Journal of Medicinal Mushrooms*.

[47] Liu W., Wang H., Pang X., Yao W., Gao X. (2010). Characterization and antioxidant activity of two low-molecular-weight polysaccharides purified from the fruiting bodies of Ganoderma lucidum. *International Journal of Biological Macromolecules*.

[48] Yuen J., Gohel M. (2008). The dual roles of Ganoderma antioxidants on urothelial cell DNA under carcinogenic attack. *Journal of Ethnopharmacology*.

[49] Mau J.-L., Lin H.-C., Chen C.-C. (2002). Antioxidant properties of several medicinal mushrooms. *Journal of Agricultural and Food Chemistry*.

[50] Kim K. C., Kim I. (1999). Ganoderma lucidum extract protects DNA from strand breakage caused by hydroxyl radical and UV irradiation. *International Journal of Molecular Medicine*.

[51] Kao P.-F., Wang S.-H., Hung W.-T., Liao Y.-H., Lin C.-M., Yang W.-B. (2012). Structural Characterization and Antioxidative Activity of Low-Molecular- Weights Beta-1,3-Glucan from the Residue of Extracted Ganoderma lucidum Fruiting Bodies. *Journal of Biomedicine and Biotechnology*.

[52] Liu Y.-J., du J. L., Cao L. P. (2015). Anti-inflammatory and hepatoprotective effects of Ganoderma lucidum polysaccharides on carbon tetrachloride-induced hepatocyte damage in common carp (Cyprinus carpio L.). *International Immunopharmacology*.

[53] Woo C. W. H., Man R. Y. K., Siow Y. L. (2005). Ganoderma lucidum inhibits inducible nitric oxide synthase expression in macrophages. *Molecular and Cellular Biochemistry*.

[54] Gill B. S., Kumar S. (2018). Ganoderic acid A targeting *β*-catenin in Wnt signaling pathway: in silico and in vitro study. *Interdisciplinary Sciences: Computational Life Sciences*.

[55] Thyagarajan A., Jiang J., Hopf A., Adamec J., Sliva D. (2006). Inhibition of oxidative stress-induced invasiveness of cancer cells by Ganoderma lucidum is mediated through the suppression of interleukin-8 secretion. *International Journal of Molecular Medicine*.

[56] Meng L.-Z., Xie J., Lv G. P. (2014). A comparative study on immunomodulatory activity of polysaccharides from two official species of Ganoderma (Lingzhi). *Nutrition and Cancer*.

[57] Ji Z., Tang Q., Zhang J., Yang Y., Jia W., Pan Y. (2007). Immunomodulation of RAW264. 7 macrophages by GLIS, a proteopolysaccharide from Ganoderma lucidum. *Journal of Ethnopharmacology*.

[58] West T. M., Wang Q., Deng B. (2019). Phosphodiesterase 5 associates with *β*2 adrenergic receptor to modulate cardiac function in type 2 diabetic hearts. *Journal of the American Heart Association*.

[59] Jacinto E., Loewith R., Schmidt A. (2004). Mammalian TOR complex 2 controls the actin cytoskeleton and is rapamycin insensitive. *Nature Cell Biology*.

[60] Porstmann T., Santos C. R., Griffiths B. (2008). SREBP activity is regulated by mTORC1 and contributes to Akt-dependent cell growth. *Cell Metabolism*.

[61] Robitaille A. M., Christen S., Shimobayashi M. (2013). Quantitative phosphoproteomics reveal mTORC1 activates de novo pyrimidine synthesis. *Science*.

[62] Michels A. A., Robitaille A. M., Buczynski-Ruchonnet D. (2010). mTORC1 directly phosphorylates and regulates human MAF1. *Molecular and Cellular Biology*.

[63] Orioli A., Praz V., Lhôte P., Hernandez N. (2016). Human MAF1 targets and represses active RNA polymerase III genes by preventing recruitment rather than inducing long-term transcriptional arrest. *Genome Research*.

[64] Holczer M., Hajdú B., Lőrincz T., Szarka A., Bánhegyi G., Kapuy O. (2019). A double negative feedback loop between mTORC1 and AMPK kinases guarantees precise autophagy induction upon cellular stress. *International Journal of Molecular Sciences*.

[65] Koren I., Reem E., Kimchi A. (2010). DAP1, a novel substrate of mTOR, negatively regulates autophagy. *Current biology*.

[66] Zhao D.-Y., Yu D. D., Ren L., Bi G. R. (2020). Ligustilide protects PC12 cells from oxygen-glucose deprivation/reoxygenation-induced apoptosis via the LKB1-AMPK-mTOR signaling pathway. *Neural Regeneration Research*.

[67] Sarbassov D. D., Ali S. M., Kim D.-H. (2004). Rictor, a novel binding partner of mTOR, defines a rapamycin-insensitive and raptor-independent pathway that regulates the cytoskeleton. *Current biology*.

[68] Shende P., Xu L., Morandi C. (2015). Cardiac mTOR complex 2 preserves ventricular function in pressure-overload hypertrophy. *Cardiovascular Research*.

[69] Olsen J. M., Sato M., Dallner O. S. (2014). Glucose uptake in brown fat cells is dependent on mTOR complex 2–promoted GLUT1 translocation. *Journal of Cell Biology*.

[70] Kim S. J., DeStefano M. A., Oh W. J. (2012). mTOR complex 2 regulates proper turnover of insulin receptor substrate-1 via the ubiquitin ligase subunit Fbw8. *Molecular Cell*.

[71] Kim H. W., Ha S. H., Lee M. N. (2010). Cyclic AMP controls mTOR through regulation of the dynamic interaction between Rheb and phosphodiesterase 4D. *Molecular and Cellular Biology*.

[72] Mátyás C., Németh B. T., Oláh A. (2017). Prevention of the development of heart failure with preserved ejection fraction by the phosphodiesterase-5A inhibitor vardenafil in rats with type 2 diabetes. *European Journal of Heart Failure*.

[73] Koser F., Loescher C., Linke W. A. (2019). Posttranslational modifications of titin from cardiac muscle: how, where, and what for?. *The FEBS Journal*.

[74] Sarnelli G., D’Alessandro A., Iuvone T. (2016). Palmitoylethanolamide modulates inflammation-associated vascular endothelial growth factor (VEGF) signaling via the Akt/mTOR pathway in a selective peroxisome proliferator-activated receptor alpha (PPAR-*α*)-dependent manner. *PLoS One*.

[75] Maile R., Stepp W. H., Eitas T., Cairns B. A. (2018). *The Mammalian Target of Rapamycin (mTOR)/Peroxisome Proliferator-Activated Receptor γ (PPARγ) axis drives immune dysfunction and outcome after burn injury*.

[76] Das A., Durrant D., Salloum F. N., Xi L., Kukreja R. C. (2015). PDE5 inhibitors as therapeutics for heart disease, diabetes and cancer. *Pharmacology & Therapeutics*.

[77] Bergmann M. W. (2010). WNT signaling in adult cardiac hypertrophy and remodeling: lessons learned from cardiac development. *Circulation Research*.

[78] Deb A. (2014). Cell–cell interaction in the heart via Wnt/*β*-catenin pathway after cardiac injury. *Cardiovascular Research*.

[79] Haybar H., Khodadi E., Shahrabi S. (2019). Wnt/*β*-catenin in ischemic myocardium: interactions and signaling pathways as a therapeutic target. *Heart Failure Reviews*.

[80] Inoki K., Ouyang H., Zhu T. (2006). TSC2 integrates Wnt and energy signals via a coordinated phosphorylation by AMPK and GSK3 to regulate cell growth. *Cell*.

[81] Aisagbonhi O., Rai M., Ryzhov S., Atria N., Feoktistov I., Hatzopoulos A. K. (2011). Experimental myocardial infarction triggers canonical Wnt signaling and endothelial-to-mesenchymal transition. *Disease Models & Mechanisms*.

[82] Ma L., Chen Z., Erdjument-Bromage H., Tempst P., Pandolfi P. P. (2005). Phosphorylation and functional inactivation of TSC2 by Erk. *Cell*.

[83] Li Y., Ha T., Gao X. (2004). NF-*κ*B activation is required for the development of cardiac hypertrophy in vivo. *American Journal of Physiology-Heart and Circulatory Physiology*.

[84] Hayden M., Ghosh S. (2004). Signaling to NF-*κ*B. *Genes & development*.

[85] Gordon J. W., Shaw J. A., Kirshenbaum L. A. (2011). Multiple facets of NF-*κ*B in the heart: to be or not to NF-*κ*B. *Circulation Research*.

[86] Harhaji Trajković L. M., Mijatović S. A., Maksimović-Ivanić D. D. (2009). Anticancer properties of Ganoderma lucidum methanol extracts in vitro and in vivo. *Nutrition and Cancer*.

[87] Sun J., He H., Xie B. J. (2004). Novel antioxidant peptides from fermented mushroom Ganoderma lucidum. *Journal of Agricultural and Food Chemistry*.

[88] Lai N.-S., Lin R.-H., Lai R.-S., Kun U.-C., Leu S.-C. (2016). Prevention of autoantibody formation and prolonged survival in New Zealand black/New Zealand white F1 mice with an ancient Chinese herb Ganoderma tsugae. *Lupus*.

[89] Zeng X., Li P., Chen X. (2019). Effects of deproteinization methods on primary structure and antioxidant activity of Ganoderma lucidum polysaccharides. *International Journal of Biological Macromolecules*.

[90] Tan X., Sun J., Xu Z. (2018). Effect of heat stress on production and in-vitro antioxidant activity of polysaccharides in Ganoderma lucidum. *Bioprocess and Biosystems Engineering*.

[91] Kumari B., Das P., Kumari R. (2016). Accelerated processing of solitary and clustered abasic site DNA damage lesions by APE1 in the presence of aqueous extract of Ganoderma lucidum. *Journal of Biosciences*.

[92] Reis F., Lima R., Morales P., Ferreira I., Vasconcelos M. (2015). Methanolic extract of Ganoderma lucidum induces autophagy of AGS human gastric tumor cells. *Molecules*.

