# Supplementary material for: Associated Targets of the Antioxidant Cardioprotection of Ganoderma lucidum in Diabetic Cardiomyopathy by Using Open Targets Platform: A Systematic Review
Source: Biomed Res Int. 2020 Jul 25;2020:7136075. doi: 10.1155/2020/7136075 (PMC7397440; doi:10.1155/2020/7136075)
Supplement: Supplementary Materials — Supplementary Table 1: association sore of 309 targets associated with diabetic cardiomyopathy in 30 recorded tissues. Supplementary Table 2: thirty-two tissue organs expressed 309 targets and types of pathways. [file 7136075.f1.docx]

Table1: Association sore of 309 targets associated with diabetic cardiomyopathy in 30 recorded tissues

|  | Target symbol | Overall AS* | datatypes Known drug AS | datatypes  literature AS | Target name |
| --- | --- | --- | --- | --- | --- |
| 1 | TRIM55 | 0.177 | 0 | 0.177 | tripartite motif containing 55 |
| 2 | PPARA | 0.117 | 0 | 0.117 | peroxisome proliferator activated receptor alpha |
| 3 | MTOR | 0.113 | 0.1 | 0.054 | mechanistic target of rapamycin kinase |
| 4 | IL6 | 0.113 | 0 | 0.113 | interleukin 6 |
| 5 | CPT1B | 0.100 | 0.1 | 0.000 | carnitine palmitoyltransferase 1B |
| 6 | CPT2 | 0.100 | 0.1 | 0.000 | carnitine palmitoyltransferase 2 |
| 7 | CXCL10 | 0.098 | 0 | 0.098 | C-X-C motif chemokine ligand 10 |
| 8 | TRIM54 | 0.081 | 0 | 0.081 | tripartite motif containing 54 |
| 9 | HMOX1 | 0.078 | 0 | 0.078 | heme oxygenase 1 |
| 10 | INS | 0.077 | 0 | 0.077 | insulin |
| 11 | NFE2L2 | 0.072 | 0 | 0.072 | nuclear factor, erythroid 2 like 2 |
| 12 | HSD11B1 | 0.070 | 0 | 0.070 | hydroxysteroid 11-beta dehydrogenase 1 |
| 13 | FGF1 | 0.070 | 0 | 0.070 | fibroblast growth factor 1 |
| 14 | CSF3 | 0.062 | 0 | 0.062 | colony stimulating factor 3 |
| 15 | BECN1 | 0.062 | 0 | 0.062 | beclin 1 |
| 16 | SIRT1 | 0.062 | 0 | 0.062 | sirtuin 1 |
| 17 | FGF21 | 0.061 | 0 | 0.061 | fibroblast growth factor 21 |
| 18 | CYP2J2 | 0.061 | 0 | 0.061 | cytochrome P450 family 2 subfamily J member 2 |
| 19 | CMA1 | 0.060 | 0 | 0.060 | chymase 1 |
| 20 | ACE2 | 0.060 | 0 | 0.060 | angiotensin I converting enzyme 2 |
| 21 | ALDH2 | 0.059 | 0 | 0.059 | aldehyde dehydrogenase 2 family member |
| 22 | GSK3B | 0.057 | 0 | 0.057 | glycogen synthase kinase 3 beta |
| 23 | NLRP3 | 0.057 | 0 | 0.057 | NLR family pyrin domain containing 3 |
| 24 | TNF | 0.057 | 0 | 0.057 | tumor necrosis factor |
| 25 | TRIM72 | 0.056 | 0 | 0.056 | tripartite motif containing 72 |
| 26 | GSN | 0.055 | 0 | 0.055 | gelsolin |
| 27 | TLR2 | 0.054 | 0 | 0.054 | toll like receptor 2 |
| 28 | PRKN | 0.054 | 0 | 0.054 | parkin RBR E3 ubiquitin protein ligase |
| 29 | APLN | 0.053 | 0 | 0.053 | apelin |
| 30 | ST3GAL4 | 0.052 | 0 | 0.052 | ST3 beta-galactoside alpha-2,3-sialyltransferase 4 |
| 31 | PPARG | 0.052 | 0 | 0.052 | peroxisome proliferator activated receptor gamma |
| 32 | FKBP1B | 0.052 | 0 | 0.052 | FKBP prolyl isomerase 1B |
| 33 | CORIN | 0.052 | 0 | 0.052 | corin, serine peptidase |
| 34 | DGUOK | 0.051 | 0 | 0.051 | deoxyguanosine kinase |
| 35 | BGLAP | 0.051 | 0 | 0.051 | bone gamma-carboxyglutamate protein |
| 36 | ALOX15 | 0.050 | 0 | 0.050 | arachidonate 15-lipoxygenase |
| 37 | TLR4 | 0.050 | 0 | 0.050 | toll like receptor 4 |
| 38 | TTN | 0.049 | 0 | 0.049 | titin |
| 39 | ANG | 0.049 | 0 | 0.049 | angiogenin |
| 40 | PRKD1 | 0.049 | 0 | 0.049 | protein kinase D1 |
| 41 | PPARGC1A | 0.048 | 0 | 0.048 | PPARG coactivator 1 alpha |
| 42 | VEGFA | 0.048 | 0 | 0.048 | vascular endothelial growth factor A |
| 43 | ADRB3 | 0.047 | 0 | 0.047 | adrenoceptor beta 3 |
| 44 | IGF1 | 0.047 | 0 | 0.047 | insulin like growth factor 1 |
| 45 | CD36 | 0.047 | 0 | 0.047 | CD36 molecule |
| 46 | TSN | 0.046 | 0 | 0.046 | translin |
| 47 | IAPP | 0.046 | 0 | 0.046 | islet amyloid polypeptide |
| 48 | IGF1R | 0.046 | 0 | 0.046 | insulin like growth factor 1 receptor |
| 49 | NOS3 | 0.046 | 0 | 0.046 | nitric oxide synthase 3 |
| 50 | PIK3CA | 0.046 | 0 | 0.046 | phosphatidylinositol-4,5-bisphosphate 3-kinase catalytic subunit alpha |
| 51 | GCH1 | 0.046 | 0 | 0.046 | GTP cyclohydrolase 1 |
| 52 | NCL | 0.045 | 0 | 0.045 | nucleolin |
| 53 | AIM2 | 0.045 | 0 | 0.045 | absent in melanoma 2 |
| 54 | APOA1 | 0.044 | 0 | 0.044 | apolipoprotein A1 |
| 55 | HSPD1 | 0.044 | 0 | 0.044 | heat shock protein family D (Hsp60) member 1 |
| 56 | PIK3CG | 0.044 | 0 | 0.044 | phosphatidylinositol-4,5-bisphosphate 3-kinase catalytic subunit gamma |
| 57 | REN | 0.043 | 0 | 0.043 | renin |
| 58 | GJA1 | 0.041 | 0 | 0.041 | gap junction protein alpha 1 |
| 59 | EDN2 | 0.041 | 0 | 0.041 | endothelin 2 |
| 60 | CASQ2 | 0.041 | 0 | 0.041 | calsequestrin 2 |
| 61 | MAPK8 | 0.041 | 0 | 0.041 | mitogen-activated protein kinase 8 |
| 62 | FGF19 | 0.041 | 0 | 0.041 | fibroblast growth factor 19 |
| 63 | DCN | 0.040 | 0 | 0.040 | decorin |
| 64 | UCN | 0.040 | 0 | 0.040 | urocortin |
| 65 | CCN2 | 0.040 | 0 | 0.040 | cellular communication network factor 2 |
| 66 | SLC33A1 | 0.040 | 0 | 0.040 | solute carrier family 33 member 1 |
| 67 | MMP2 | 0.040 | 0 | 0.040 | matrix metallopeptidase 2 |
| 68 | MAPK3 | 0.040 | 0 | 0.040 | mitogen-activated protein kinase 3 |
| 69 | POSTN | 0.039 | 0 | 0.039 | periostin |
| 70 | OSM | 0.039 | 0 | 0.039 | oncostatin M |
| 71 | FGF2 | 0.039 | 0 | 0.039 | fibroblast growth factor 2 |
| 72 | BCL6 | 0.039 | 0 | 0.039 | BCL6 transcription repressor |
| 73 | ATP6AP2 | 0.039 | 0 | 0.039 | ATPase H+ transporting accessory protein 2 |
| 74 | EPO | 0.039 | 0 | 0.039 | erythropoietin |
| 75 | EPHB2 | 0.038 | 0 | 0.038 | EPH receptor B2 |
| 76 | TAX1BP1 | 0.038 | 0 | 0.038 | Tax1 binding protein 1 |
| 77 | SLC2A4 | 0.038 | 0 | 0.038 | solute carrier family 2 member 4 |
| 78 | MME | 0.038 | 0 | 0.038 | membrane metalloendopeptidase |
| 79 | HMGB1 | 0.038 | 0 | 0.038 | high mobility group box 1 |
| 80 | MICU1 | 0.037 | 0 | 0.037 | mitochondrial calcium uptake 1 |
| 81 | UTS2 | 0.037 | 0 | 0.037 | urotensin 2 |
| 82 | ROCK2 | 0.037 | 0 | 0.037 | Rho associated coiled-coil containing protein kinase 2 |
| 83 | SERPINA12 | 0.037 | 0 | 0.037 | serpin family A member 12 |
| 84 | NOX4 | 0.036 | 0 | 0.036 | NADPH oxidase 4 |
| 85 | SRI | 0.036 | 0 | 0.036 | sorcin |
| 86 | MAPK9 | 0.036 | 0 | 0.036 | mitogen-activated protein kinase 9 |
| 87 | IGF2 | 0.036 | 0 | 0.036 | insulin like growth factor 2 |
| 88 | CXCR4 | 0.036 | 0 | 0.036 | C-X-C motif chemokine receptor 4 |
| 89 | AGTR2 | 0.036 | 0 | 0.036 | angiotensin II receptor type 2 |
| 90 | LPL | 0.036 | 0 | 0.036 | lipoprotein lipase |
| 91 | INSR | 0.035 | 0 | 0.035 | insulin receptor |
| 92 | ANGPT1 | 0.035 | 0 | 0.035 | angiopoietin 1 |
| 93 | CNR1 | 0.035 | 0 | 0.035 | cannabinoid receptor 1 |
| 94 | IL33 | 0.035 | 0 | 0.035 | interleukin 33 |
| 95 | CAV3 | 0.034 | 0 | 0.034 | caveolin 3 |
| 96 | PAEP | 0.034 | 0 | 0.034 | progestagen associated endometrial protein |
| 97 | KDM2B | 0.034 | 0 | 0.034 | lysine demethylase 2B |
| 98 | RASGRF1 | 0.034 | 0 | 0.034 | Ras protein specific guanine nucleotide releasing factor 1 |
| 99 | ACE | 0.034 | 0 | 0.034 | angiotensin I converting enzyme |
| 100 | PNPLA2 | 0.034 | 0 | 0.034 | patatin like phospholipase domain containing 2 |
| 101 | ATF6 | 0.034 | 0 | 0.034 | activating transcription factor 6 |
| 102 | NOD2 | 0.034 | 0 | 0.034 | nucleotide binding oligomerization domain containing 2 |
| 103 | CRP | 0.034 | 0 | 0.034 | C-reactive protein |
| 104 | NR1H4 | 0.034 | 0 | 0.034 | nuclear receptor subfamily 1 group H member 4 |
| 105 | RETN | 0.033 | 0 | 0.033 | resistin |
| 106 | HIF1A | 0.033 | 0 | 0.033 | hypoxia inducible factor 1 subunit alpha |
| 107 | C1QTNF3 | 0.033 | 0 | 0.033 | C1q and TNF related 3 |
| 108 | ATP2A2 | 0.033 | 0 | 0.033 | ATPase sarcoplasmic/endoplasmic reticulum Ca2+ transporting 2 |
| 109 | KLK1 | 0.032 | 0 | 0.032 | kallikrein 1 |
| 110 | ELMO1 | 0.032 | 0 | 0.032 | engulfment and cell motility 1 |
| 111 | GCK | 0.032 | 0 | 0.032 | glucokinase |
| 112 | DDAH2 | 0.032 | 0 | 0.032 | dimethylarginine dimethylaminohydrolase 2 |
| 113 | SYK | 0.032 | 0 | 0.032 | spleen associated tyrosine kinase |
| 114 | XPR1 | 0.032 | 0 | 0.032 | xenotropic and polytropic retrovirus receptor 1 |
| 115 | VEGFB | 0.032 | 0 | 0.032 | vascular endothelial growth factor B |
| 116 | PDE5A | 0.031 | 0 | 0.031 | phosphodiesterase 5A |
| 117 | CNR2 | 0.031 | 0 | 0.031 | cannabinoid receptor 2 |
| 118 | HDAC3 | 0.031 | 0 | 0.031 | histone deacetylase 3 |
| 119 | MAPKAPK2 | 0.031 | 0 | 0.031 | MAPK activated protein kinase 2 |
| 120 | HSPE1 | 0.031 | 0 | 0.031 | heat shock protein family E (Hsp10) member 1 |
| 121 | SIRT2 | 0.031 | 0 | 0.031 | sirtuin 2 |
| 122 | SOAT1 | 0.031 | 0 | 0.031 | sterol O-acyltransferase 1 |
| 123 | NRG1 | 0.030 | 0 | 0.030 | neuregulin 1 |
| 124 | DIRAS3 | 0.030 | 0 | 0.030 | DIRAS family GTPase 3 |
| 125 | SMAD3 | 0.030 | 0 | 0.030 | SMAD family member 3 |
| 126 | DUSP5 | 0.030 | 0 | 0.030 | dual specificity phosphatase 5 |
| 127 | KLF4 | 0.030 | 0 | 0.030 | Kruppel like factor 4 |
| 128 | XIAP | 0.030 | 0 | 0.030 | X-linked inhibitor of apoptosis |
| 129 | RYR2 | 0.029 | 0 | 0.029 | ryanodine receptor 2 |
| 130 | PHB | 0.029 | 0 | 0.029 | prohibitin |
| 131 | TRPC3 | 0.029 | 0 | 0.029 | transient receptor potential cation channel subfamily C member 3 |
| 132 | IL1B | 0.028 | 0 | 0.028 | interleukin 1 beta |
| 133 | ESRRG | 0.028 | 0 | 0.028 | estrogen related receptor gamma |
| 134 | NEB | 0.028 | 0 | 0.028 | nebulin |
| 135 | PRDX3 | 0.028 | 0 | 0.028 | peroxiredoxin 3 |
| 136 | SETD2 | 0.028 | 0 | 0.028 | SET domain containing 2, histone lysine methyltransferase |
| 137 | ADRB2 | 0.028 | 0 | 0.028 | adrenoceptor beta 2 |
| 138 | SLC9A1 | 0.028 | 0 | 0.028 | solute carrier family 9 member A1 |
| 139 | CAT | 0.028 | 0 | 0.028 | catalase |
| 140 | EGFR | 0.028 | 0 | 0.028 | epidermal growth factor receptor |
| 141 | PRKCA | 0.027 | 0 | 0.027 | protein kinase C alpha |
| 142 | ESR1 | 0.027 | 0 | 0.027 | estrogen receptor 1 |
| 143 | TGM2 | 0.027 | 0 | 0.027 | transglutaminase 2 |
| 144 | PARP1 | 0.027 | 0 | 0.027 | poly(ADP-ribose) polymerase 1 |
| 145 | IRS1 | 0.027 | 0 | 0.027 | insulin receptor substrate 1 |
| 146 | TXNIP | 0.026 | 0 | 0.026 | thioredoxin interacting protein |
| 147 | VDAC1 | 0.026 | 0 | 0.026 | voltage dependent anion channel 1 |
| 148 | OGA | 0.026 | 0 | 0.026 | O-GlcNAcase |
| 149 | AKT1 | 0.025 | 0 | 0.025 | AKT serine/threonine kinase 1 |
| 150 | MEF2A | 0.025 | 0 | 0.025 | myocyte enhancer factor 2A |
| 151 | DUSP1 | 0.025 | 0 | 0.025 | dual specificity phosphatase 1 |
| 152 | MSC | 0.025 | 0 | 0.025 | musculin |
| 153 | CXCL8 | 0.024 | 0 | 0.024 | C-X-C motif chemokine ligand 8 |
| 154 | DGKZ | 0.024 | 0 | 0.024 | diacylglycerol kinase zeta |
| 155 | OGT | 0.024 | 0 | 0.024 | O-linked N-acetylglucosamine (GlcNAc) transferase |
| 156 | DAPK2 | 0.024 | 0 | 0.024 | death associated protein kinase 2 |
| 157 | MALT1 | 0.023 | 0 | 0.023 | MALT1 paracaspase |
| 158 | SLC25A4 | 0.023 | 0 | 0.023 | solute carrier family 25 member 4 |
| 159 | CELF1 | 0.023 | 0 | 0.023 | CUGBP Elav-like family member 1 |
| 160 | SMAD7 | 0.023 | 0 | 0.023 | SMAD family member 7 |
| 161 | NPPA | 0.023 | 0 | 0.023 | natriuretic peptide A |
| 162 | SCT | 0.023 | 0 | 0.023 | secretin |
| 163 | PPARD | 0.023 | 0 | 0.023 | peroxisome proliferator activated receptor delta |
| 164 | CCDC47 | 0.022 | 0 | 0.022 | coiled-coil domain containing 47 |
| 165 | LIPE | 0.022 | 0 | 0.022 | lipase E, hormone sensitive type |
| 166 | RLN3 | 0.022 | 0 | 0.022 | relaxin 3 |
| 167 | SPP1 | 0.022 | 0 | 0.022 | secreted phosphoprotein 1 |
| 168 | BDKRB1 | 0.022 | 0 | 0.022 | bradykinin receptor B1 |
| 169 | TLX2 | 0.022 | 0 | 0.022 | T cell leukemia homeobox 2 |
| 170 | LEP | 0.022 | 0 | 0.022 | leptin |
| 171 | ZGLP1 | 0.022 | 0 | 0.022 | zinc finger GATA like protein 1 |
| 172 | VDR | 0.021 | 0 | 0.021 | vitamin D receptor |
| 173 | ARSA | 0.021 | 0 | 0.021 | arylsulfatase A |
| 174 | NOS2 | 0.021 | 0 | 0.021 | nitric oxide synthase 2 |
| 175 | NR3C2 | 0.021 | 0 | 0.021 | nuclear receptor subfamily 3 group C member 2 |
| 176 | GDF15 | 0.021 | 0 | 0.021 | growth differentiation factor 15 |
| 177 | CASP3 | 0.021 | 0 | 0.021 | caspase 3 |
| 178 | SIRT3 | 0.021 | 0 | 0.021 | sirtuin 3 |
| 179 | HNF1A | 0.020 | 0 | 0.020 | HNF1 homeobox A |
| 180 | HDAC4 | 0.020 | 0 | 0.020 | histone deacetylase 4 |
| 181 | ANPEP | 0.020 | 0 | 0.020 | alanyl aminopeptidase, membrane |
| 182 | DDIT4 | 0.020 | 0 | 0.020 | DNA damage inducible transcript 4 |
| 183 | PRKCB | 0.020 | 0 | 0.020 | protein kinase C beta |
| 184 | PLG | 0.020 | 0 | 0.020 | plasminogen |
| 185 | SLC17A5 | 0.020 | 0 | 0.020 | solute carrier family 17 member 5 |
| 186 | SPIN1 | 0.020 | 0 | 0.020 | spindlin 1 |
| 187 | SERPINE1 | 0.020 | 0 | 0.020 | serpin family E member 1 |
| 188 | TACR1 | 0.020 | 0 | 0.020 | tachykinin receptor 1 |
| 189 | RBFOX2 | 0.020 | 0 | 0.020 | RNA binding fox-1 homolog 2 |
| 190 | TRPV1 | 0.019 | 0 | 0.019 | transient receptor potential cation channel subfamily V member 1 |
| 191 | FABP4 | 0.019 | 0 | 0.019 | fatty acid binding protein 4 |
| 192 | KCNH2 | 0.019 | 0 | 0.019 | potassium voltage-gated channel subfamily H member 2 |
| 193 | CADM1 | 0.019 | 0 | 0.019 | cell adhesion molecule 1 |
| 194 | MTDH | 0.018 | 0 | 0.018 | metadherin |
| 195 | PRCP | 0.018 | 0 | 0.018 | prolylcarboxypeptidase |
| 196 | TFAM | 0.018 | 0 | 0.018 | transcription factor A, mitochondrial |
| 197 | NOD1 | 0.018 | 0 | 0.018 | nucleotide binding oligomerization domain containing 1 |
| 198 | CASR | 0.018 | 0 | 0.018 | calcium sensing receptor |
| 199 | DPP4 | 0.018 | 0 | 0.018 | dipeptidyl peptidase 4 |
| 200 | BSCL2 | 0.018 | 0 | 0.018 | BSCL2 lipid droplet biogenesis associated, seipin |
| 201 | ATF3 | 0.018 | 0 | 0.018 | activating transcription factor 3 |
| 202 | VIP | 0.018 | 0 | 0.018 | vasoactive intestinal peptide |
| 203 | EGLN3 | 0.018 | 0 | 0.018 | egl-9 family hypoxia inducible factor 3 |
| 204 | FN1 | 0.018 | 0 | 0.018 | fibronectin 1 |
| 205 | IL17A | 0.018 | 0 | 0.018 | interleukin 17A |
| 206 | EDN1 | 0.018 | 0 | 0.018 | endothelin 1 |
| 207 | CCL2 | 0.018 | 0 | 0.018 | C-C motif chemokine ligand 2 |
| 208 | S100A12 | 0.018 | 0 | 0.018 | S100 calcium binding protein A12 |
| 209 | SLC5A1 | 0.018 | 0 | 0.018 | solute carrier family 5 member 1 |
| 210 | FGL2 | 0.017 | 0 | 0.017 | fibrinogen like 2 |
| 211 | SCO2 | 0.017 | 0 | 0.017 | SCO cytochrome c oxidase assembly protein 2 |
| 212 | MAOA | 0.017 | 0 | 0.017 | monoamine oxidase A |
| 213 | ENHO | 0.017 | 0 | 0.017 | energy homeostasis associated |
| 214 | TP53 | 0.017 | 0 | 0.017 | tumor protein p53 |
| 215 | NPY | 0.017 | 0 | 0.017 | neuropeptide Y |
| 216 | TRPA1 | 0.017 | 0 | 0.017 | transient receptor potential cation channel subfamily A member 1 |
| 217 | S1PR1 | 0.017 | 0 | 0.017 | sphingosine-1-phosphate receptor 1 |
| 218 | STAT3 | 0.017 | 0 | 0.017 | signal transducer and activator of transcription 3 |
| 219 | TLR3 | 0.017 | 0 | 0.017 | toll like receptor 3 |
| 220 | TRIM63 | 0.017 | 0 | 0.017 | tripartite motif containing 63 |
| 221 | MDM2 | 0.017 | 0 | 0.017 | MDM2 proto-oncogene |
| 222 | PITX1 | 0.017 | 0 | 0.017 | paired like homeodomain 1 |
| 223 | TIMP2 | 0.017 | 0 | 0.017 | TIMP metallopeptidase inhibitor 2 |
| 224 | NGF | 0.017 | 0 | 0.017 | nerve growth factor |
| 225 | BCL2 | 0.016 | 0 | 0.016 | BCL2 apoptosis regulator |
| 226 | RXFP1 | 0.016 | 0 | 0.016 | relaxin family peptide receptor 1 |
| 227 | CERS2 | 0.016 | 0 | 0.016 | ceramide synthase 2 |
| 228 | ENO1 | 0.016 | 0 | 0.016 | enolase 1 |
| 229 | MIF | 0.016 | 0 | 0.016 | macrophage migration inhibitory factor |
| 230 | CTH | 0.016 | 0 | 0.016 | cystathionine gamma-lyase |
| 231 | RXFP3 | 0.016 | 0 | 0.016 | relaxin family peptide receptor 3 |
| 232 | NPR2 | 0.016 | 0 | 0.016 | natriuretic peptide receptor 2 |
| 233 | CDKN1A | 0.016 | 0 | 0.016 | cyclin dependent kinase inhibitor 1A |
| 234 | CEACAM6 | 0.016 | 0 | 0.016 | CEA cell adhesion molecule 6 |
| 235 | CSE1L | 0.016 | 0 | 0.016 | chromosome segregation 1 like |
| 236 | CSF3R | 0.016 | 0 | 0.016 | colony stimulating factor 3 receptor |
| 237 | CTSD | 0.016 | 0 | 0.016 | cathepsin D |
| 238 | SLC11A2 | 0.016 | 0 | 0.016 | solute carrier family 11 member 2 |
| 239 | MMP9 | 0.016 | 0 | 0.016 | matrix metallopeptidase 9 |
| 240 | NPS | 0.015 | 0 | 0.015 | neuropeptide S |
| 241 | THBS1 | 0.015 | 0 | 0.015 | thrombospondin 1 |
| 242 | KDR | 0.015 | 0 | 0.015 | kinase insert domain receptor |
| 243 | STK11 | 0.015 | 0 | 0.015 | serine/threonine kinase 11 |
| 244 | PDC | 0.015 | 0 | 0.015 | phosducin |
| 245 | ENO3 | 0.015 | 0 | 0.015 | enolase 3 |
| 246 | GSDMD | 0.015 | 0 | 0.015 | gasdermin D |
| 247 | PTGS1 | 0.015 | 0 | 0.015 | prostaglandin-endoperoxide synthase 1 |
| 248 | GRK2 | 0.015 | 0 | 0.015 | G protein-coupled receptor kinase 2 |
| 249 | GPRC6A | 0.015 | 0 | 0.015 | G protein-coupled receptor class C group 6 member A |
| 250 | CYCS | 0.015 | 0 | 0.015 | cytochrome c, somatic |
| 251 | KLKB1 | 0.015 | 0 | 0.015 | kallikrein B1 |
| 252 | TIMP4 | 0.015 | 0 | 0.015 | TIMP metallopeptidase inhibitor 4 |
| 253 | RASA1 | 0.015 | 0 | 0.015 | RAS p21 protein activator 1 |
| 254 | CERS5 | 0.015 | 0 | 0.015 | ceramide synthase 5 |
| 255 | RAC1 | 0.015 | 0 | 0.015 | Rac family small GTPase 1 |
| 256 | TGFB3 | 0.015 | 0 | 0.015 | transforming growth factor beta 3 |
| 257 | SLC2A1 | 0.015 | 0 | 0.015 | solute carrier family 2 member 1 |
| 258 | ZBTB16 | 0.015 | 0 | 0.015 | zinc finger and BTB domain containing 16 |
| 259 | COL1A1 | 0.015 | 0 | 0.015 | collagen type I alpha 1 chain |
| 260 | SMS | 0.015 | 0 | 0.015 | spermine synthase |
| 261 | XBP1 | 0.015 | 0 | 0.015 | X-box binding protein 1 |
| 262 | PSME1 | 0.015 | 0 | 0.015 | proteasome activator subunit 1 |
| 263 | MAP3K5 | 0.014 | 0 | 0.014 | mitogen-activated protein kinase kinase kinase 5 |
| 264 | EDNRA | 0.014 | 0 | 0.014 | endothelin receptor type A |
| 265 | CCN1 | 0.014 | 0 | 0.014 | cellular communication network factor 1 |
| 266 | IL10 | 0.014 | 0 | 0.014 | interleukin 10 |
| 267 | LGALS3 | 0.014 | 0 | 0.014 | galectin 3 |
| 268 | APOE | 0.014 | 0 | 0.014 | apolipoprotein E |
| 269 | LDLR | 0.014 | 0 | 0.014 | low density lipoprotein receptor |
| 270 | SPARC | 0.014 | 0 | 0.014 | secreted protein acidic and cysteine rich |
| 271 | GLP1R | 0.014 | 0 | 0.014 | glucagon like peptide 1 receptor |
| 272 | IFNG | 0.014 | 0 | 0.014 | interferon gamma |
| 273 | ACD | 0.014 | 0 | 0.014 | ACD shelterin complex subunit and telomerase recruitment factor |
| 274 | CST3 | 0.014 | 0 | 0.014 | cystatin C |
| 275 | ICAM1 | 0.014 | 0 | 0.014 | intercellular adhesion molecule 1 |
| 276 | SCTR | 0.014 | 0 | 0.014 | secretin receptor |
| 277 | NFATC3 | 0.014 | 0 | 0.014 | nuclear factor of activated T cells 3 |
| 278 | ELN | 0.014 | 0 | 0.014 | elastin |
| 279 | TNC | 0.014 | 0 | 0.014 | tenascin C |
| 280 | MAT2B | 0.014 | 0 | 0.014 | methionine adenosyltransferase 2B |
| 281 | CREBBP | 0.014 | 0 | 0.014 | CREB binding protein |
| 282 | FOXO1 | 0.014 | 0 | 0.014 | forkhead box O1 |
| 283 | PINK1 | 0.014 | 0 | 0.014 | PTEN induced kinase 1 |
| 284 | CAST | 0.014 | 0 | 0.014 | calpastatin |
| 285 | AIF1 | 0.012 | 0 | 0.012 | allograft inflammatory factor 1 |
| 286 | SMAD2 | 0.012 | 0 | 0.012 | SMAD family member 2 |
| 287 | CEBPB | 0.012 | 0 | 0.012 | CCAAT enhancer binding protein beta |
| 288 | SULT1E1 | 0.012 | 0 | 0.012 | sulfotransferase family 1E member 1 |
| 289 | ACOT1 | 0.012 | 0 | 0.012 | acyl-CoA thioesterase 1 |
| 290 | MED13 | 0.011 | 0 | 0.011 | mediator complex subunit 13 |
| 291 | PTGS2 | 0.011 | 0 | 0.011 | prostaglandin-endoperoxide synthase 2 |
| 292 | ARC | 0.010 | 0 | 0.010 | activity regulated cytoskeleton associated protein |
| 293 | GPBAR1 | 0.010 | 0 | 0.010 | G protein-coupled bile acid receptor 1 |
| 294 | ANXA1 | 0.010 | 0 | 0.010 | annexin A1 |
| 295 | SOD2 | 0.010 | 0 | 0.010 | superoxide dismutase 2 |
| 296 | GZMH | 0.010 | 0 | 0.010 | granzyme H |
| 297 | CASP1 | 0.010 | 0 | 0.010 | caspase 1 |
| 298 | NCF1 | 0.009 | 0 | 0.009 | neutrophil cytosolic factor 1 |
| 299 | FASLG | 0.009 | 0 | 0.009 | Fas ligand |
| 300 | OXTR | 0.008 | 0 | 0.008 | oxytocin receptor |
| 301 | APOL2 | 0.008 | 0 | 0.008 | apolipoprotein L2 |
| 302 | NPPB | 0.008 | 0 | 0.008 | natriuretic peptide B |
| 303 | LEPR | 0.008 | 0 | 0.008 | leptin receptor |
| 304 | SRF | 0.008 | 0 | 0.008 | serum response factor |
| 305 | HSPB3 | 0.007 | 0 | 0.007 | heat shock protein family B (small) member 3 |
| 306 | AGTR1 | 0.007 | 0 | 0.007 | angiotensin II receptor type 1 |
| 307 | PPP5C | 0.007 | 0 | 0.007 | protein phosphatase 5 catalytic subunit |
| 308 | IARS1 | 0.004 | 0 | 0.004 | isoleucyl-tRNA synthetase 1 |
| 309 | ABHD5 | 0.004 | 0 | 0.004 | abhydrolase domain containing 5 |

*AS= association score.

Table 2: Thirty two tissue organs expressed 309 targets and types of pathways

|  | Tissue organs expressed 309 targets | pathway types (targets number) |
| --- | --- | --- |
|  | 1. Bladder organ 2. Blood 3. Brain 4. Breast 5. Colon 6. Connective tissue 7. Endocrine gland 8. Esophagogastric junction 9. Esophagus 10. Exocrine gland 11. Heart 12. Immune organ 13. Intestine 14. Kidney 15. Liver 16. Lung 17. Mucosa 18. Musculature 19. Nerve 20. Oral gland 21. Pancreas 22. Peritoneum 23. Pharynx 24. Rectum 25. Reproductive organ 26. Reproductive structure 27. Skeletal element 28. Skin of body 29. Spinal cord 30. Spleen 31. Stomach 32. Vasculature | Signal Transduction (63)  Immune System (47)  Metabolism of proteins (39)  Metabolism (31)  Gene expression (Transcription)(25)  Hemostasis (23)  Disease (22)  Developmental Biology (20)  Extracellular matrix organization (18)  Cellular responses to external stimuli (14)  Transport of small molecules (11)  Muscle contraction (11)  Vesicle-mediated transport(10)  Organelle biogenesis and maintenance (4)  Programmed Cell Death (4)  Autophagy (4)  Neuronal System (3)  Cell Cycle (3)  Circadian Clock (3) |
|  |  |  |
